# Ion-Exchange Treatment of Perfluorinated Carboxylic Acids in Water: Comparison of Polystyrenic and Polyacrylic Resin Structures and Impact of Sulfate on Their Performance

**DOI:** 10.1021/acsestwater.1c00501

**Published:** 2022-06-22

**Authors:** M. Feisal Rahman, William B. Anderson, Sigrid Peldszus, Peter M. Huck

**Affiliations:** † Department of Civil and Environmental Engineering, University of Waterloo, 200 University Avenue West, Waterloo, Ontario N2L 3G1, Canada; ‡ Living Deltas Hub, Dept. of Geography and Environmental Sciences, Northumbria University, Newcastle-upon-Tyne, NE1 8ST, United Kingdom

**Keywords:** drinking water, ion exchange, natural organic matter, perfluorinated carboxylic acid (PFCA) removal, per- and polyfluoroalkyl substance (PFAS) removal, sulfate

## Abstract

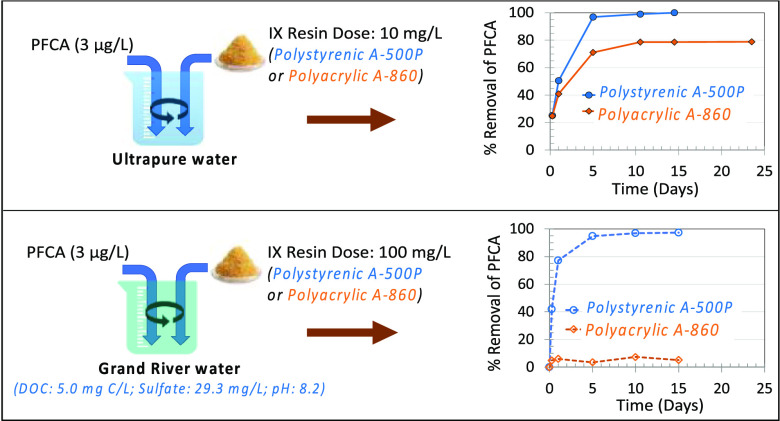

The removal of three perfluorinated carboxylic acids (PFCAs)—PFHpA, PFOA, and PFNA—in ultrapure and river water was evaluated using two anion-exchange resins—previously unreported macroporous polystyrenic A-500P and a more widely studied macroporous polyacrylic A-860. Both resins had similar properties, allowing direct comparison of PFCA removal performance between the two resin structures/matrices. This study also presents a new gas chromatography–mass spectrometry (GC/MS) method developed for PFCA analysis in water. In ultrapure water, A-500P exhibited higher removal capacity and faster removal compared to A-860, suggesting greater effectiveness of the polystyrenic structure compared to the polyacrylic structure. In the Grand River water, the target PFCAs were well removed by A-500P but not A-860. However, both resins achieved similarly high overall reductions of dissolved organic carbon (∼75%), suggesting, later confirmed in ultrapure water experiments, that inorganic anions (sulfate particularly) were the dominant competitors for the A-860 resin. The uncharged styrenic and acrylic beads (base materials) of the two tested resins were unable to remove PFOA, implying that the dominant removal mechanism involves charge interactions between the negatively charged PFCA and the positively charged anion-exchange functional groups.

## Abstract 2

PFCA removal in ultrapure and river water using IX resins demonstrated superior performance of the tested polystyrenic resin to the polyacrylic resin, and the presence of sulfate more severely affected the performance of the latter.

## 1.0 Introduction

Per- and polyfluoroalkyl substances (PFASs) are an emerging class of drinking water contaminants that have been detected globally at trace concentrations in drinking water.^1−4^ Due to their widespread occurrence, long half-life in human tissues, and potential human health impacts,^5−7^ several PFAS-related regulations or guidelines for drinking water are currently in force or are being considered in various jurisdictions.^5^ For example, Health Canada guidelines for maximum acceptable concentrations (MACs) for perfluorooctanoic acid (PFOA) and perfluorosulfonic acid (PFOS) in drinking water are 0.2 and 0.6 μg/L, based on exposure solely to PFOA and PFOS, respectively.^8,9^ On the other hand, the USEPA made final determinations to regulate PFOA and PFOS, confirming that it will move forward with the process to propose and promulgate a national primary drinking water regulation for the two contaminants under the Safe Drinking Water Act of 1996.^10^

PFASs have been reported to be not amenable to a variety of drinking water treatment processes including conventional coagulation–flocculation, biofiltration, ozonation, and even advanced oxidation processes.^2,3,11,12^ On the other hand, advanced treatment processes such as tight membrane filtration and activated carbon adsorption have been reported to be effective in removing PFASs from drinking water.^3,13,14^ In addition to these processes, ion-exchange (IX) resin treatment is being considered as a potential alternative for the removal of PFASs. Available bench-scale studies corroborate the promise of anion-exchange resins for the removal of PFASs from drinking water.^15−23^

Recent studies also reported simultaneous removal of PFASs, natural organic matter (NOM), and inorganic anions from natural water using anion IX resins. However, both NOM and inorganic anions, especially owing to their higher concentration in water (∼mg/L) as opposed to that of the PFAS (ng/L to μg/L) would exert competition for removal sites.^19,20^ A recent review paper^24^ on PFAS removal by IX treatment concluded that “any demonstration of IX treatment efficacy for PFASs must examine the role of NOM and inorganic ions on the efficacy of PFAS uptake in natural waters. With the exception of few studies, most of the available data focuses on the PFAS removal in deionized waters or synthetic waters with standard isolated NOM fractions”. In addition, investigation is also needed to understand the effect of the resin matrix on PFAS removal and elucidate the effect of electrostatic interaction as opposed to hydrophobic interaction during PFAS removal using ion-exchange resins.

This study was undertaken to provide new insights on the use of IX resins for the removal of the target PFCAs while experiencing competition from NOM. In doing so, the primary objective of the study was to elucidate the effectiveness of two ion-exchange resins for the removal of three PFASs—perfluoroheptanoic acid (PFHpA), PFOA, and perfluorononanoic acid (PFNA)—from ultrapure and natural water at environmentally relevant concentrations. All three target compounds are perfluorinated carboxylic acids (PFCAs), a sub-group of PFASs, and have been widely reported in natural water and finished drinking water.^3^

The study was designed to use two IX resins which have similar properties (Table 1) but differ in the resin backbone/matrix, thereby enabling a direct comparison of the impact of the IX resin structure (polyacrylic vs polystyrenic) on PFCA removal. Moreover, the study investigated the currently underreported direct competitive effect of background water quality parameters (namely NOM and inorganic anions) on PFCA removal using IX treatment. The dominant removal mechanisms for the selected PFCAs by IX resins were also studied; that is, the role of electrostatic versus hydrophobic interactions was elucidated by comparing the performance of resins with and without ion-exchange sites (i.e., uncharged resin beads/resin base material). Apart from these, two additional novel aspects of the study are (i) PFCA removal performance of the A-500P resin, which has not been reported to date in the literature compared to the well-studied A860; and (ii) a new method for PFCA analysis involving more widely accessible but less commonly adopted gas chromatography–mass spectrometry (GC/MS) instrumentation [as opposed to liquid chromatography–mass spectrometry (LC/MS)].

**Table 1:** Properties of the Anion-Exchange Resins and Resin Beads

resin/bead	matrix	capacity (Cl-form) (equiv/L)^a^	functional group^a^	moisture content (%)^b^	particle size range (mm)^a^	SBET (m^2^/g)^+^	pore volume (cm^3^/g)
A-860	macroporous polyacrylic	0.8	quaternary ammonium (trimethyl ammonium) (type-I)	67.7	0.3–1.2	<1	could not be measured
A-500P	macroporous polystyrenic	0.8	quaternary ammonium (trimethyl ammonium) (type-I)	68.0	0.425–1.2	4.06	0.021
acrylic beads	macroporous polyacrylic	N/A	none	48.3	N/A	9	0.044
styrenic beads	macroporous polystyrenic	N/A	none	36.7	N/A	<1	could not be measured

a Data from the manufacturer; N/A-not available.

b Determined by drying resin beads in an oven at 105 °C for 24 h; moisture content of the beads was determined on bead samples that were washed with 200 bed volumes of UPW; + SBET-Brunauer–Emmett–Teller (BET) specific surface area; and SBET analysis of the resins and resin beads was conducted at a certified commercial laboratory (Quantachrome Laboratory, Florida, US).

## 2.0 Materials and Methods

### 2.1 Target Compounds

PFHpA (99%), PFOA (96%), and PFNA (97%) were purchased from Sigma-Aldrich (St. Louis, MO, USA). Molecular structures and the physicochemical properties for each of the selected target compounds are provided in the Supporting Information (SI) (Table SI-1). Except for the mass-labeled internal standard (^13^C_8_–PFOA), all target PFCAs were obtained as solids, and stock solutions of individual PFCAs were prepared at a concentration of 1000 mg/L in methanol and stored at 4 °C. Working standards of PFCA mixtures or individual PFCAs were prepared by diluting stock solutions appropriately to either 10 or 1 mg/L and were also kept refrigerated at 4 °C. Prepared solutions (both stock solutions and working standards) were stored for no longer than 9 months in the refrigerator. Note that the stock solutions for spiking in the resin experiments were prepared in ultrapure water (UPW) (see Section 2.3).

### 2.2 Resins and Uncharged Resin Beads

Two organic scavenging strong-base anion-exchange resins from Purolite—macroporous polystyrenic A-500P and macroporous acrylic A-860 (Purolite, Bala Cynwyd, PA)—were selected for the study. Both ion-exchange resins were used as received without further treatment. Base materials of the two resins, the uncharged resin beads (polyacrylic and polystyrenic resin beads), were donated by Purolite Canada. These uncharged beads were washed with 200 bed volumes of UPW to remove fines and organics in which they were stored or produced. Typically, styrenic resins and styrenic beads are more hydrophobic, while acrylic resins and acrylic beads are more hydrophilic in nature. The resin beads are uncharged, while both ion-exchange resins have quaternary ammonium groups as their anion-exchange functional groups. The exact compositions of these functional groups are proprietary.

PFAS removal efficacy of A-500P has not yet been reported in the published literature, while A-860 has been studied previously.^18−20,25^ Except for the resin matrix, both anion-exchange resins have similar properties (Table 1) which facilitate direct comparison of PFCA removal effectiveness between polyacrylic and polystyrenic IX resins. Laura del Moral et al.^25^ previously compared polystyrenic A-520E and polyacrylic A-860, both of which possess strong-base quaternary ammonium functional groups and a macroporous structure. However, the resins differ in the nature of the quaternary ammonium groups, as A520E has triethyl ammonium functional groups (total capacity, 0.9 equiv/L), whereas A860 has trimethyl ammonium functional groups (total capacity, 0.8 equiv/L). They^25^ noted that “although polymer composition was the focus of resin properties in this research, the differing functional groups between A520E and A860 do present a confounding factor”. The choice of resins used for the current study, however, was able to overcome this confounding factor as both resins possess trimethyl ammonium functional groups and have the same capacity and other similar properties. Both resins were from the same manufacturer and were also used in practice as organic scavengers.

It is noteworthy to mention that A-500P has been discontinued and replaced with A-500Plus, which has the same chemical composition but a different bead size (information obtained through email communication with Mr. Don Downey from Purolite, Canada). Hence, even though A-500P has been discontinued, the findings of the current study are still relevant.

### 2.3 Waters

UPW (18.2 MΩ) generated from a Millipore Milli-Q UV Plus system (Mississauga, ON) was used throughout the study. Dissolved organic carbon (DOC) levels in the UPW were always below 0.3 mg C/L, and pH values ranged between 4.9 and 6.1.

Raw (untreated) Grand River water (GRW) (Southern Ontario, Canada) was collected from the intake of a drinking water treatment plant located on this river. Two batches of GRW were collected for the study, and none of the target PFCAs were detected in these batches. The first batch was collected on February 3, 2014, spiked, and then used to conduct the experiments with the selected adsorbents (set 1 experiments). The second batch was collected on May 9, 2014, and the second set of experiments (set 2 experiments) was conducted to confirm the trends observed. Properties of the two batches of GRW are listed in Table SI-2. The river water had already gone through initial screening. No further alterations were made to the collected raw waters except that they were stored overnight in the laboratory at 4 °C (thus allowing precipitation of some of the particulate matter) before being spiked with the target PFCA. Prior to the experiments, the spiked water was carefully poured off to exclude the settled particulates.

No pH adjustments were conducted during this study. Untreated GRW pH values were 8.2 and 8.5, respectively, for set 1 and set 2 (reported in Table SI-2). UPW pH ranged from 5.4 to 5.9. The pH of the water did not change during the experiments. The target PFCAs are strongly acidic (estimated p*K*_a_ < 1) and are expected to be in anionic form in the pH ranges of ultrapure and surface water.^5,26^ Stock solutions of the target PFCA used for resin experiments were prepared in UPW at a concentration of 10 mg/L without any organic solvent and stored for a maximum of 9 months at 4 °C. Throughout the study, UPW and GRW were spiked as required using the stock prepared in UPW. Following a spike with the target PFCAs, the water was allowed to equilibrate overnight prior to starting kinetic experiments. The individual nominal compound target spike concentration was 3.0 μg/L in all tests. The actual spiked concentrations were measured at the beginning of each experiment.

### 2.4 Experimental Approach

To illustrate and compare the PFCA removal performance of the two selected resins, kinetic and isotherm experiments were conducted as described below. The test protocols/experimental conditions such as resin doses and initial PFCA concentrations used in the current study are comparable to previously published studies (see Table SI-3). Three types of controls, namely pure blanks (no PFCA, no resin), spiked blanks (negative control, i.e., spiked PFCA, no resin), and treatment blanks (no PFCA, resin added), were used for both UPW and GRW experiments.

#### 2.4.1 Kinetic Experiments

Bottle point adsorption kinetic experiments with the selected resins and resin beads were conducted in 1 L polypropylene opaque bottles (VWR, West Chester, PA) at 150 rpm on an orbital shaker (Barnstead/Thermolyne, Dubuque, IA). For kinetics experiments in UPW, wet resins equivalent to 10 mg dry weight of resin were added to 1 L of spiked water solution containing PFCAs. For GRW kinetics experiments, 1 L of spiked surface water was poured into each sample bottle and wet resins equivalent to 100 mg dry weight of resin were added. The higher dosage of IX resins in GRW as opposed to UPW was used to anticipate direct competition from the natural water matrix.

Both UPW and GRW were spiked with a mixture of PFCAs and with only PFOA in some selected experiments (termed PFOA only). Spiked raw water blanks were also monitored for potential PFCA degradation and contamination. Sample bottles were then taken off the shaker at different time intervals and processed to monitor the time-dependent removal of the spiked contaminants. All experiments were conducted at room temperature (∼20 °C) to minimize the effect of temperature change on adsorption. To differentiate the effect of sorption of the PFAS onto particulate matter, spiked blanks (negative control—PFCA added, no resin) were used for UPW water and the GRW experiments. No reduction in PFCA concentration was observed in the spiked blanks.

A pseudo-second-order model developed by Ho^27^ has been widely used to quantitatively describe adsorption kinetics.^28,29^ The rate law for the pseudo-second-order model can be described as follows (eq 1)
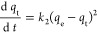
1where *k*_2_ (mg ng^–1^ d^–1^) is the rate constant for adsorption, *q*_e_ (ng mg^–1^) is the total amount adsorbed at equilibrium, and *q*_t_ (ng mg^–1^) is the amount adsorbed at time *t* (d).

Integrating eq 1 for the range within the boundary conditions *t* = 0 to *t* = *t* and *q*_t_ = 0 to *q*_t_ = *q*_t_ provides the expression for sorption kinetics as follows (eq 2)
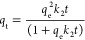
2

Equation 2 can be rearranged to obtain eq 3
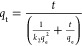
3

The pseudo-second-order model can be expressed in a linearized form as in eq 4. The initial sorption rate ϑ (ng mg^–1^ d^–1^) reflects kinetic performance and is expressed in eq 5. The last two equations will be used to describe PFAS removal kinetics in UPW and GRW using the tested resins.
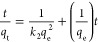
4
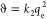
5

#### 2.4.2 Isotherm Experiments

For isotherm experiments (to determine the adsorptive capacity of the resins), different amounts (dry weights ranging from 0.5 to 12 mg) of the tested resins were added to 1 L of UPW solution. All isotherm experiments were conducted with single solutes at a target nominal concentration of 3 μg/L. Samples were then stirred for the time to reach removal equilibrium as was determined during the UPW kinetic experiments (10 days).

Various models are used to describe isotherms. However, the Freundlich isotherm model is most frequently used in water treatment practice^30^ and, as such, was used in the current study as well. The linear form of the Freundlich model is expressed as below (eq 6)
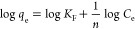
6where *q*_e_ is the equilibrium solid phase concentration (ng/mg), *C*_e_ is the equilibrium liquid phase concentration, and *K*_F_ and 1/*n* are Freundlich parameters. Experimental data were fitted to the model using Microsoft Excel.

### 2.5 Analysis

Analyses of the target compounds in water samples were performed by GC/MS preceded by solid phase extraction (SPE) and derivatization. The target PFCAs were derivatized using butanol in the presence of sulfuric acid and heat. By employing a central composite factorial design, the optimum derivatization reaction conditions were established. The method detection limits (MDLs) were 11–30 ng/L in UPW and 16–49 ng/L in GRW water, depending on the target compound. In order to achieve these low detection limits, the entire 1 L volume of a sample bottle from the isotherm/kinetic experiments was required for the SPE. This also made it necessary to have one bottle for each data point. GC/MS method performance parameters are listed in Table SI-4. Brief details of the GC/MS analytical method can be found in the Supporting Information (SI-C), and the detailed analytical method development has been described by Rahman.^31^

It should be noted that PFASs are typically analyzed by LC/MS as opposed to GC/MS. A new GC/MS method was developed for the purpose of this study as we did not have access to an LC/MS at the time of this study. The developed GC/MS method remains a novel aspect of this study. While it can be argued that LC/MS is the technology of choice, the existence of a GC/MS method can open the world of PFAS testing and research to analytical laboratory users who cannot afford to purchase an LC/MS or have sufficient funds to support off-site PFAS analyses. Having said that, using the GC/MS-based method imposed several limitations, for example, perfluoroalkyl sulfonates (PFSA) including PFOS were not amenable to the developed GC/MS method. Also, the performance of the method for small-chain PFCAs (<C6) was poor. Hence, the target compounds of the study were limited to three long-chain PFCAs (PFHpA, PFOA, and PFNA), which at that point in time (2013–2015) were being considered under USEPA’s third unregulated contaminant monitoring rule, UCMR3. Furthermore, sample preparation for the method owing to the SPE and derivatization process took much longer compared to sample preparation for LC–MS which does not require these steps. This also limited the number of samples that could be analyzed during each batch of experiments. The study therefore, instead of using replicates, repeated experiments in both UPW and GRW to confirm the PFCA removal trends using the tested IX resins.

The DOC content of the UPW was measured using a wet oxidation OI Analytical model 1010 TIC-TOC analyzer (College Station, TX). The oxidizing agent was 100 g/L Na_2_S_2_O_8_. The samples were initially preserved by lowering the pH to 2–3 using 1 N H_3_PO_4_. The instrument was calibrated using standard solutions of potassium biphthalate (C_8_H_5_KO_4_) at appropriate concentrations to measure low DOC levels in UPW.

The injection volume was 5 mL, and three replicates of each sample were processed. NOM fractions (humic substances, biopolymers (BPs), and building blocks (BB)) were measured by liquid chromatography with organic carbon detection (LC–OCD) (DOC Labor Dr. Huber, Karlsruhe, Germany).^32^ UV_254_ absorbance was measured with a UV–vis spectrometer (Cary 100, Agilent Technologies, Mississauga, ON), and specific UV absorbance (SUVA) was calculated as follows
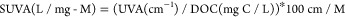


Sample pH was measured using an Orion 720A pH meter (Boston, MA), and conductivity was measured with a Mandel conductivity meter (Weilheim, Germany). Inorganic anions were analyzed with a Dionex AS-DV ion chromatography system (Thermo Scientific) using standard ASTM test methods for anions in water (ASTM Designation D4327-11).

## 3.0 Results and Discussion

### 3.1 Kinetics of PFCA Removal

**Figure 1:**
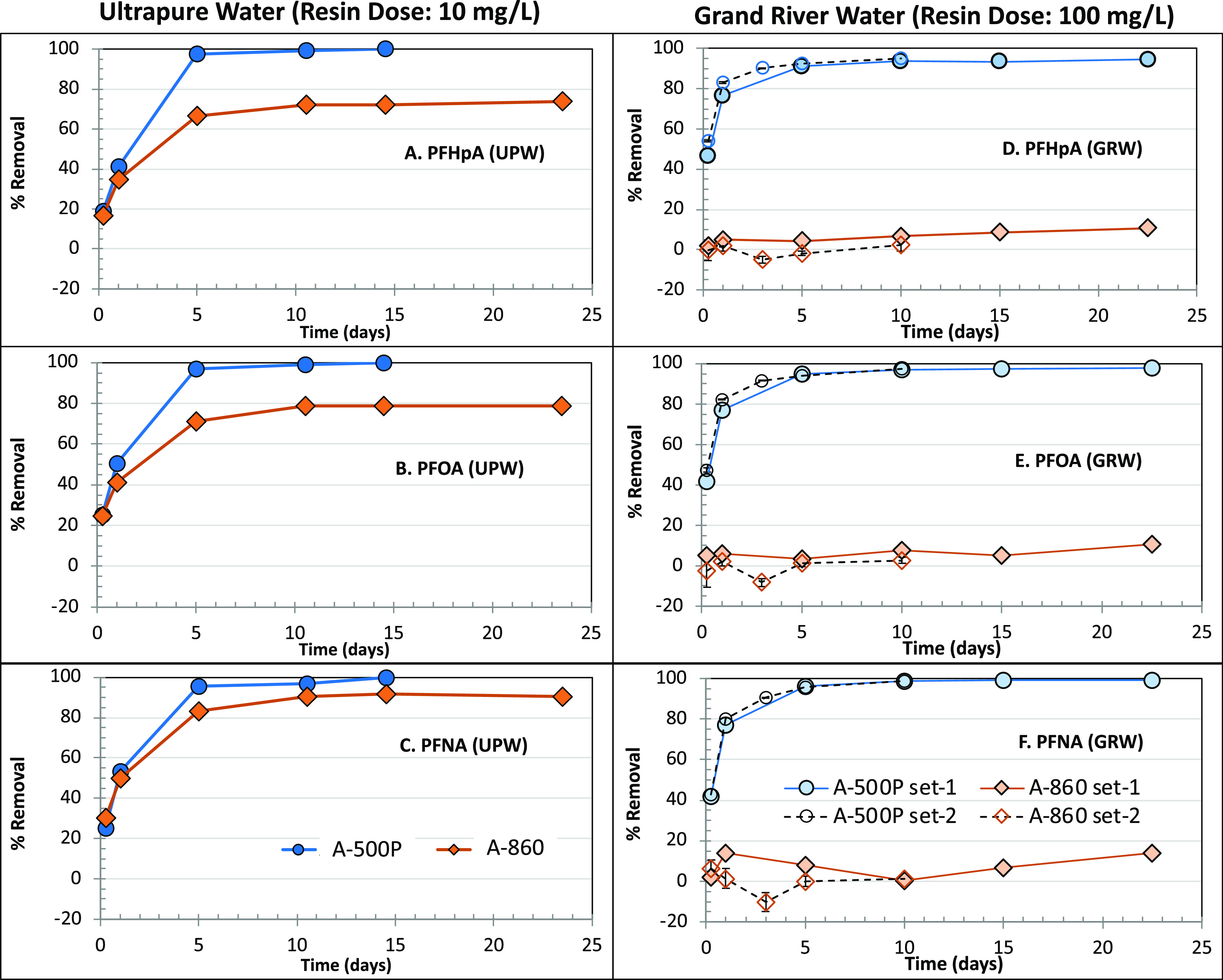
Removal of target PFCAs as a function of time in UPW (panels A–C) and GRW (panels D–F). Experimental conditions: target nominal spiked PFAS concentration was 3 μg/L, and all three target PFASs were spiked simultaneously; pH was not adjusted in UPW or GRW. The resin dose was 10 mg/L in UPW and 100 mg/L in GRW; in GRW, set 1 experiments were conducted using water collected on February 3, 2014 (DOC: 5 mg C/L; pH: 8.2; sulfate: 29.3 mg/L), and set 2 experiments used water collected on May 9, 2014 (DOC: 4.7 mg C/L; pH: 8.5; sulfate: 16.6 mg/L). Error bars in set2 experiments indicate the maximum and minimum removals of two replicate analyses (i.e., two sample bottles per data point for the set2 experiments). Removal of PFCAs by IX resins in GRW illustrated the reproducibility of removal trends.

The removal kinetics of the target PFCAs in both UPW and GRW are presented in Figure 1. In UPW at a resin dose of 10 mg/L, the polystyrenic anion-exchange resin A-500P exhibited higher removal and faster kinetics for all three target PFCAs (Figure 1A–C) compared to the polyacrylic A-860 resin. In GRW at a resin dose of 100 mg/L, A-500P was able to achieve greater than 93% removal of the target PFCA, while A-860 exhibited less than 15% removal (set 1 experiments in Figure 1D–F). Higher removal of PFCAs by polystyrenic resins as opposed to polyacrylic resins in both waters is also in line with other studies.^15,18,24,25,33^ Experiments in natural water were repeated using a different batch of water (set 2 experiments in Figure 1D–F), which confirmed the reproducibility of the removal trends for both resins.

The fitted pseudo-second-order model parameters (expressed in mass-base units), including the corresponding correlation coefficients along with the experimentally derived equilibrium adsorption amounts, are presented in Table 2 (model parameter values are expressed in molar base units, and 95% confidence interval values of the parameters are presented in Tables SI-5 and SI-6, respectively). As seen from the pseudo-second-order rate constants listed, A-500P exhibited superior kinetics in both UPW and GRW, suggesting better performance of the polystyrenic structure compared to the polyacrylic structure. While the removal rates in UPW were comparable between the two tested resins, in GRW the removal rate was considerably higher for A-500P. The poor removal of PFCAs by A-860 resin in GRW explains the poor fit of the pseudo-second-order model, in particular for PFNA.

**Table 2:** PseudoSecond-Order Kinetics Model Parameters in UPW and GRW for the Target PFCAs for Mixed-Solute Experiments^a^

	PFHpA		PFOA		PFNA		*k*_2_ (mg ng–^1^ d–^1^)			ϑ (ng mg–^1^ d–^1^)			*R*^2^		
resins	*q*_e_ (ng/mg)	exp. *q*_e_ (ng/mg)	*q*_e_ (ng/mg)	exp. *q*_e_ (ng/mg)	*q*_e_ (ng/mg)	exp. *q*_e_ (ng/mg)	PFHpA	PFOA	PFNA	PFHpA	PFOA	PFNA	PFHpA	PFOA	PFNA
UPW (resin dose: 10 mg/L; individual nominal target PFCA concentration: 3000 ng/L)															
A-500P	418	371	397	362	403	371	0.0020	0.0028	0.0031	357	435	500	0.99	0.99	0.99
A-860	288	276	303	290	357	347	0.0037	0.0047	0.0046	303	435	588	0.99	0.99	0.99
GRW (resin dose: 100 mg/L; individual nominal target PFCA concentration: 3000 ng/L)															
A-500P	39	38	35	35	36	35	0.096	0.093	0.095	143	116	120	0.99	1.00	1.00
A-860	5	4	3	4	2	5	0.057	0.085	0.027	1.2	1.0	0.1	0.89	0.71	0.02

a Exp. *q*_e_: experimental *q*_e_.

It is noteworthy to mention that the parameters of the pseudo-second-order model are dependent on the resin/contaminant ratio.^18,34^ This study did not attempt to establish a resin dose for actual treatment conditions but rather intended to understand and compare the performance of the two ion-exchange resins. Given the experimental conditions used herein (the relatively low resin doses and the PFAS level higher than expected in natural waters), the kinetic parameters reported here should be interpreted with this in mind in considering full-scale/actual treatment conditions.

Although the polyacrylic anion-exchange resin A-860 achieved 73–95% removal of the target PFCA in UPW at a resin dose of 10 mg/L, it failed to achieve any substantial removal of the target PFCA in GRW even though the resin dose was 100 mg/L. Dixit et al.^19^ observed less than 30% removal of PFOA PFOA (initial concentration 500 ng/L) by A-860 at a 50 mg/L resin dose in the presence of both NOM and inorganic anions in Eagle Lake water (DOC: 3 mg C/L; sulfate 5 mg/L; bicarbonate 4.5 mg/L). When the resin dose was increased to 1000 mg/L, A-860 resin was able to achieve complete removal of PFOA simultaneously with NOM and inorganic anions in the Eagle Lake water. At a resin dose of 50 mg/L, A-860, however, exhibited lower removal of PFOA in Eagle Lake water containing both NOM and inorganic anions, compared to water containing only NOM (Suwannee River NOM 5 mg/L), indicating detrimental impact of inorganic anions on PFCA removal by IX resins.^19^ GRW contained inorganic anions (in addition to DOC at 4.7–5.0 mg C/L), and the sulfate concentration (Table SI-2) was higher than that reported for Eagle Lake water. The collective competition exerted for A-860 resin sites likely explains the loss of PFCA removal capacity in natural water observed during the current study. However, Liu,^35^ while observing a loss in PFAS capacity of A-860 in their study with synthetic water having groundwater as a background matrix, did not observe as drastic a loss of PFAS removal capacity of A-860. Their study at DOC: 9.3 mg/L, sulfate: 94 mg/L, and an initial PFOA concentration of 298 μg/L resulted in about 40% removal of PFOA after 24 h (see Table SI-7). Liu^35^ using a different set of IX resins also reported loss of removal capacity for PFASs in GRW owing to competition from inorganic anions and NOM. The impact of inorganic anions on PFCA removal by both A-860 and A-500P is discussed in detail in Section 3.4.

### 3.2 Isotherms

**Figure 2:**
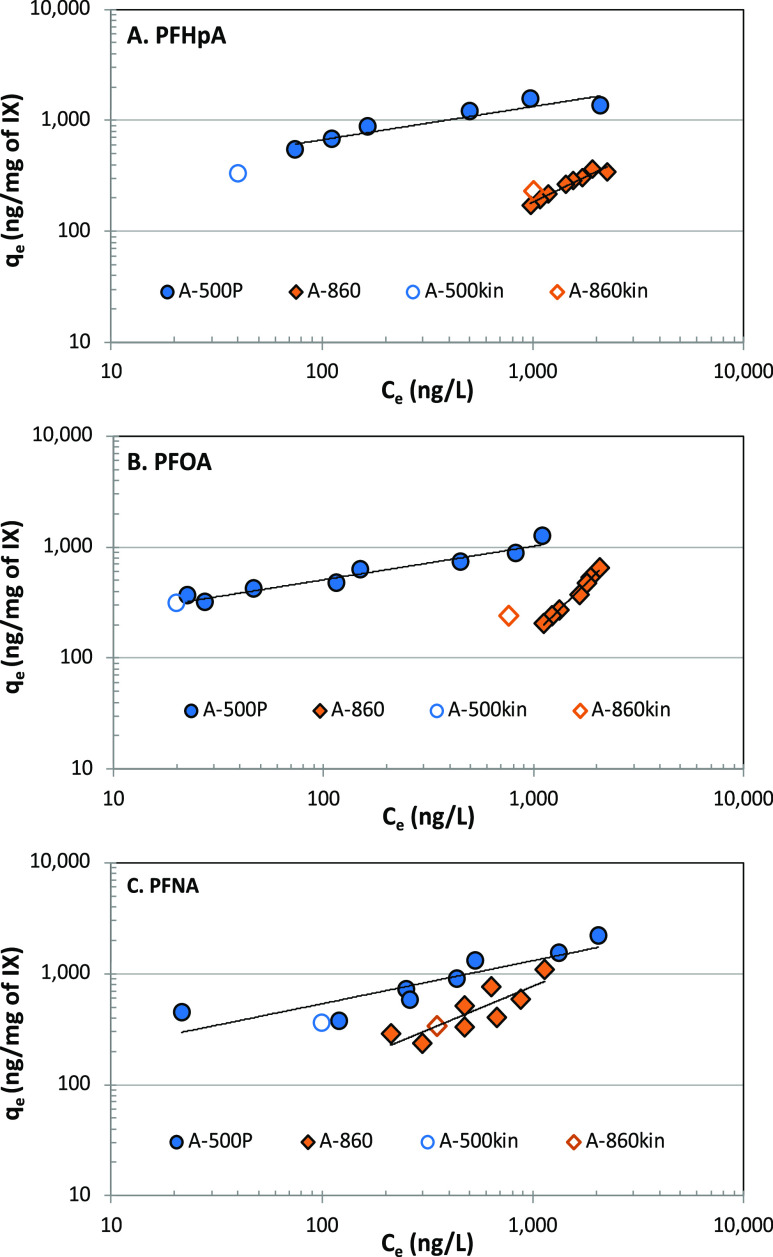
Single-solute adsorption isotherms in UPW on two IX resins for the three target PFCAs: (A) PFHpA, (B) PFOA, and (C) PFNA. kin = kinetic experiment data points at equilibrium.

Single-solute Freundlich adsorption isotherms for A-860 and A-500P in UPW for the three target PFCAs are presented in Figure 2. As with the kinetic experiments, A-500P resin exhibited a higher removal capacity for all three target PFCAs compared to that of A-860. It is also evident that for A-500P and A-860, adsorption data at equilibrium during kinetics experiments are similar to those for the obtained isotherms (except for the PFOA data for A-860), indicating that adsorption trends at equilibrium obtained from both types of experiments are similar, as would be expected. The deviation of the PFOA data for A-860 (Figure 2) may have been due to the removal behavior reflected in the high 1/*n* value as well as the narrow equilibrium liquid phase concentration range observed with this specific isotherm (see Table SI-8).

### 3.3 Removal of NOM and NOM Fractions

**Figure 3:**
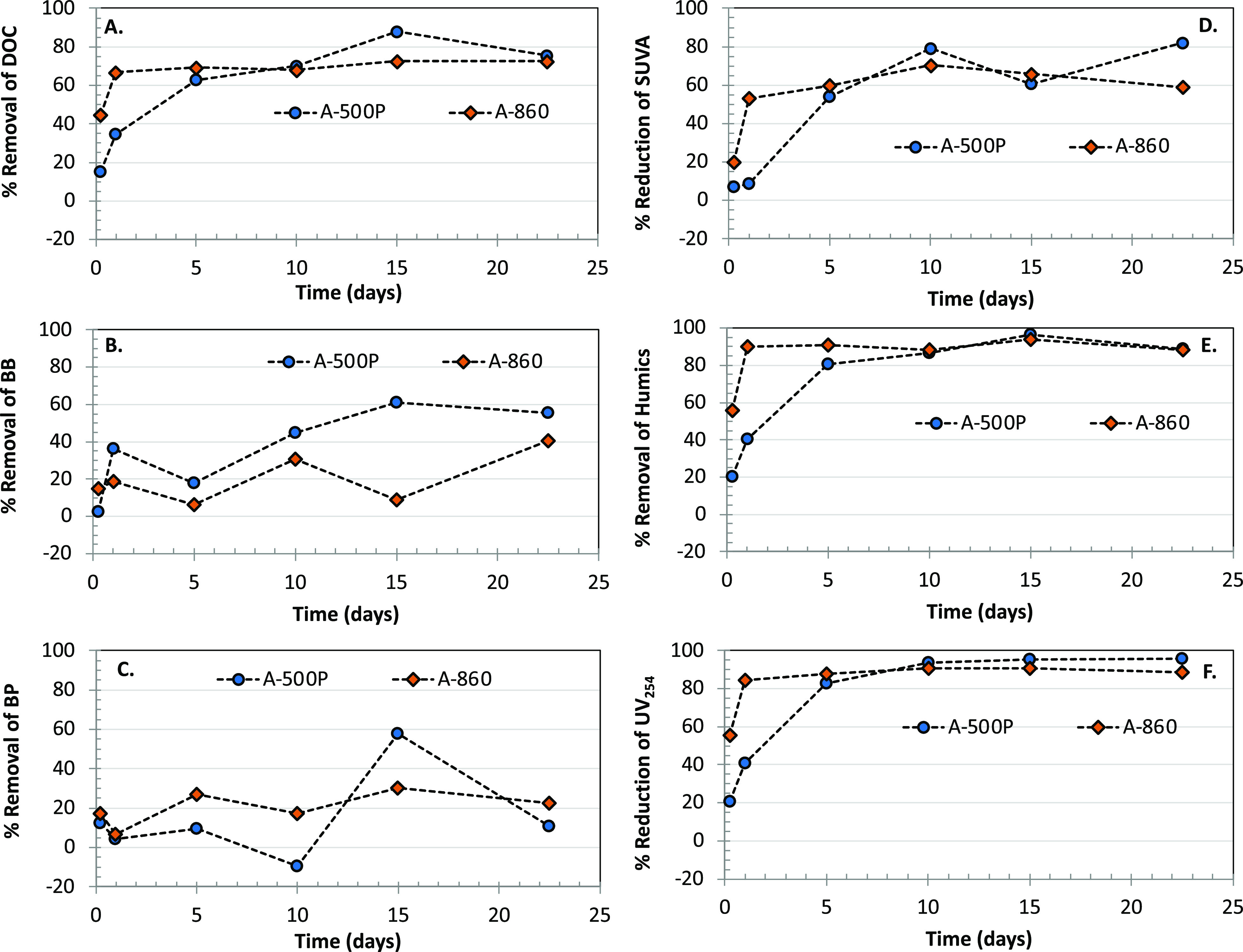
Removal of DOC and select DOC fractions in GRW (set 1 experiments) over time; (A) DOC, (B) BP, (C) BB, (D) SUVA, (E) humics, (F) UV254; DOC—5.0 mg C/L, humics—3.6 mg C/L, BP—0.25 mg C/L, BB—0.65 mg C/L; and IX resin dose of 100 mg/L.

The selected anion-exchange resins are marketed as organic scavengers and can achieve high DOC removals (Purolite, 2006). LC–OCD analysis of the PFCA-spiked raw and treated GRW (set 1 experiments) revealed that at a resin dose of 100 mg/L, both anion-exchange resins achieved nearly 75% removal of the DOC present in GRW within 10 days of contact time, and the removal did not improve substantially even after an additional 12 days of contact (Figure 3A). As can be seen in Figure 3, polyacrylic A-860, being hydrophilic, removed DOC faster than polystyrenic A-500P. Reproducibility of DOC and DOC fraction removal trends using the two resins was confirmed during set 2 experiments, which used a different batch of GRW (Figure SI-1). Removal of DOC observed during this study was comparable to that in previous studies^18,25^ that used A-860 resin (see Table SI-7). Dixit et al.^18^ reported that at a resin dose of 1000 mg/L or above, A-860 resin was able to achieve ∼90% removal of NOM.

NOM removal during ion exchange is primarily based on electrostatic interactions between negatively charged functional groups in the NOM and ion-exchange sites, rather than physical adsorption.^36,37^ Cornelissen et al.^36^ commented that during the ion-exchange treatment, physical adsorption may incidentally occur but is “neither an effective nor controllable mechanism compared to the primary mechanism”. It has also been reported that inorganic anions, in particular sulfate, may be an important determinant of DOC removal in natural water compared to other inorganic anions such as bicarbonate, nitrate, and bromide.^38^

The removal of various DOC fractions of GRW by the selected IX resins is illustrated in Figure 3B–F. The anion-exchange resins preferentially removed humics compared to other measured LC–OCD fractions, as illustrated by over 90% removal of this dominant DOC fraction in GRW (Figure 3E). Similar preferential removal of humics has been observed by others as well.^36,39^ Of the two resins, polyacrylic A-860 resin, perhaps owing to its hydrophilic structure, more rapidly removed humics compared to the polystyrenic A-500P resin. Of the two resins, A-500P appeared to remove higher concentrations of BBs compared to A-860 (Figure 3B), while the latter resin generally achieved higher removal of BPs (Figure 3C). However, the overall removal of BP was low (<35%). The scatter in BP percentage reduction is likely due to their generally low initial concentrations. Others have reported low effectiveness of anion-exchange resins in removing BPs from water as well.^36^

SUVA at 254 nm, which is used as a surrogate parameter for the aromatic content of NOM, was also substantially (∼60%) decreased following anion-exchange resin treatment (Figure 3B). Such decreases in SUVA in GRW indicate that the DOC composition of GRW is considerably altered, that is, more hydrophilic, following treatment with the two selected anion-exchange resins. Preferential removal of aromatic fractions of DOC by IX resins has been observed by other previous studies as well.^16,40^

A-500P has a high equilibrium PFCA removal capacity (Figure 1B), while the DOC removal kinetics with A-860 are substantially faster compared to A-500P (Figure 3A). Such trends indicate that A-860 could potentially be used as a pre-treatment step for an A-500P-type resin (for example: Purolite A-500Plus; note: Purolite A-500P is no longer manufactured) or in a mixture with an A-500P-type resin in natural water and thereby reduce direct competition from inorganic anions and NOM for anion-exchange sites on the A-500P type resin, leading to improved removal efficiency for the PFCAs. Future studies could thus investigate whether combining the anion-exchange resins with activated carbon treatment or even combining the two types of resins can enhance overall PFCA removal in natural water.

### 3.4 Effect of Inorganic Anions on PFAS Removal

As discussed previously, PFCA removal capacities of both resins, in particular A-860, were substantially decreased in GRW, indicating the negative impact of the natural water matrix during treatment with IX resins. Studies by Dixit and colleagues^18−20^ reported that the background water matrix, more specifically the charge density and molecular weight distribution of source water NOM and inorganic anions, affected PFAS uptake during IX treatment. Arevalo Perez^21^ and Laura del Moral et al.^25^ noted that DOC has relatively less of an adverse impact on PFAS removal by ion exchange compared to the ionic strength of water (i.e., the concentration of ions present). Substantial differences in PFCA removal and similar DOC removal by the two tested resins (both having similar capacity) in natural water observed during the current study also indicate that for the tested experimental conditions, inorganic anions may have been the dominant competitors for the target PFCA removal.

In GRW, the A-860 resin achieved somewhat higher removal of sulfate compared to A-500P, while A-500P achieved better nitrate removal (Figure SI-2). Thus, it can be postulated that the loss of PFCA removal capacity of A-860 probably resulted from the competition exerted by the high concentration of sulfate present in GRW.

**Figure 4:**
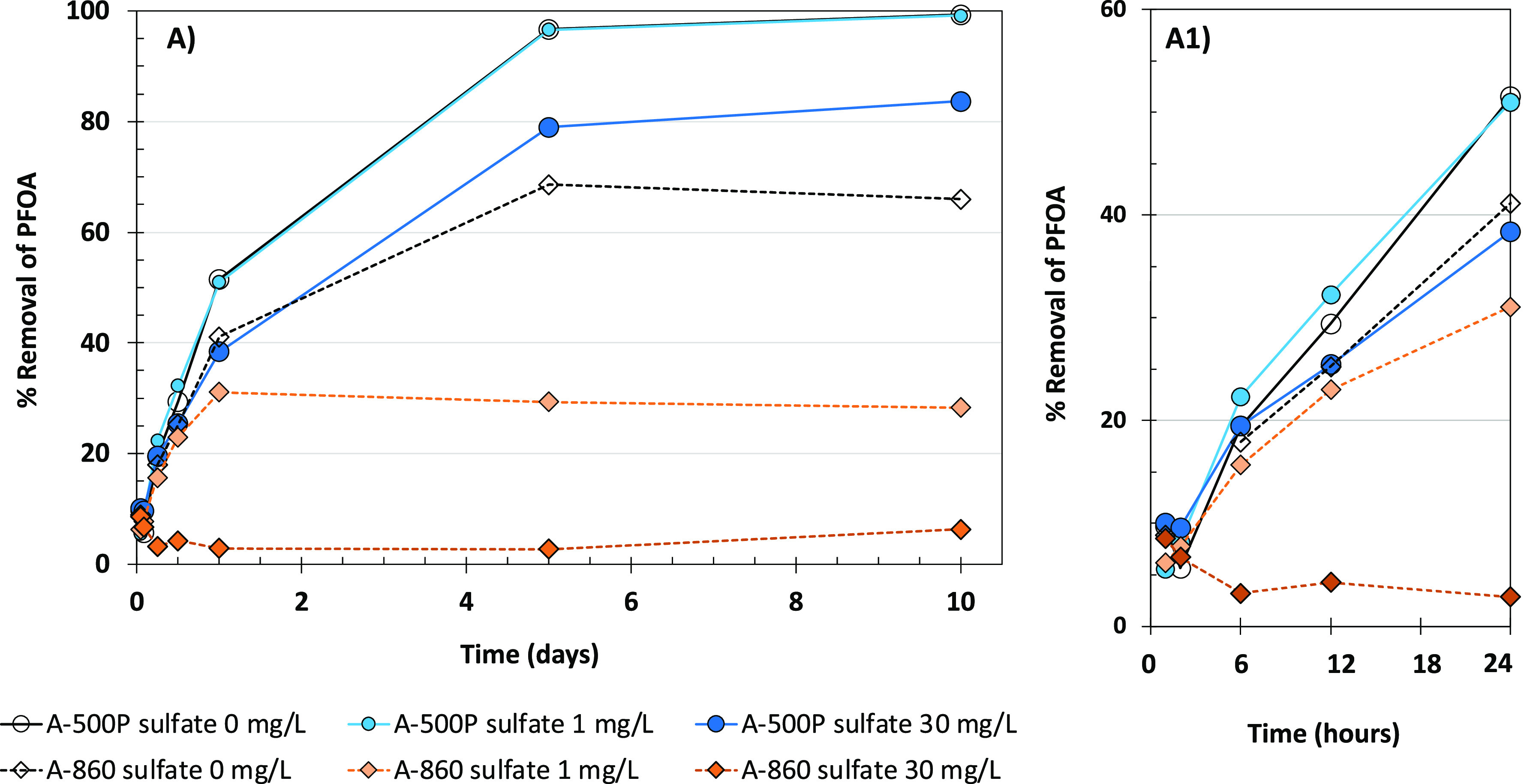
Effect of sulfate on PFOA removal kinetics in UPW; resin dose = 10 mg/L; plot A1 provides enhanced resolution of the initial data points shown in plot A; only PFOA was spiked in UPW; pH not adjusted.

Subsequent experiments in UPW spiked with 1 and 30 mg/L sulfate confirmed that increasing sulfate concentration more severely affected PFAS removal capacities for A-860 than for A-500P (Figure 4). The higher sulfate dose of 30 mg/L was chosen as the GRW sample used during set 1 experiments contained 29.3 mg/L of sulfate. The study is intended to confirm if the removal trends in UPW match those in GRW in the presence of a similar level of sulfate. Dixit et al. (2020) also conducted their experiments in synthetic and natural water that contained similar levels of sulfate. The lower sulfate dose of 1 mg/L was chosen to see the impact of a relatively small amount of sulfate on removal performance of the two resins in UPW.

In UPW, in the presence of 30 mg/L of sulfate and at a resin dose of 10 mg/L, A-860 nearly completely lost its PFOA removal capacity, while for A-500P PFOA, removal decreased only by about 20% compared to UPW without sulfate. This confirmed the higher sulfate selectivity of A-860 over A-500P. Liu^35^ using a different set of IX resins also concluded that sulfate as opposed to nitrate had a more detrimental impact on PFAS removal in GRW.

Since natural water matrices will vary depending on their location and source, the PFCA removal trends observed during the current study will not universally apply to other natural water matrices. Dixit et al.^19^ reported that A-860 at a resin dose of 1000 mg/L was able to achieve complete removal of a suite of PFASs (initial concentration 500 ng/L) from Eagle Lake water, while the removal was less than 30% in the same water when the resin dose was lowered to 50 mg/L. Nonetheless, if sulfate is present, particularly at elevated concentrations, utilities considering treatment of PFASs should consider this effect carefully with respect to using A-860-type IX resins.

### 3.5 Removal Mechanism Using the Ion-Exchange Resin

**Figure 5:**
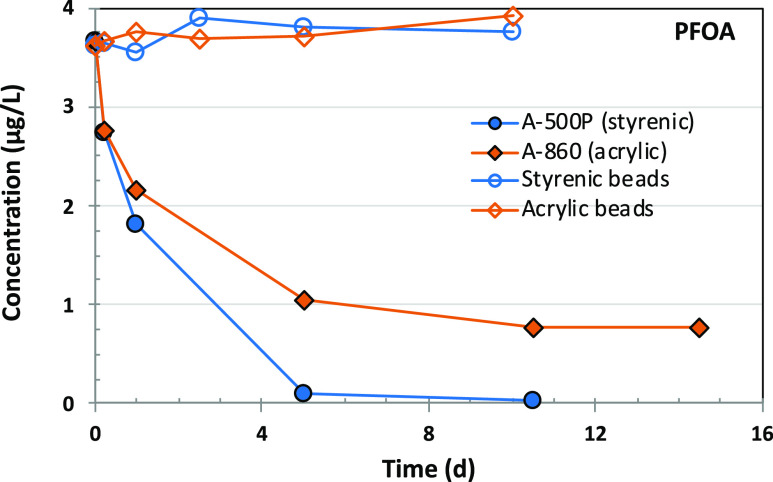
PFOA removal in UPW as a function of time using ion-exchange resins and uncharged resin base materials termed as resin beads (e.g., styrenic beads are the base material for A-500P, while acrylic beads are for A-860). Experimental conditions: resin/beads dose: 10 mg/L; target nominal spiked PFAS concentration was 3 μg/L, and all three target PFASs were spiked simultaneously; pH was not adjusted in UPW or GRW.

PFCAs can be removed by two possible mechanisms during IX treatment: (a) ion-exchange (electrostatic interaction between the anionic functional group of the PFCA and the cationic functional group on the anion-exchange resin) and (b) adsorption (hydrophobic interactions between the polymer backbone of the IX resin and the hydrophobic PFAS chain).^23^ To investigate the contributions of hydrophobic interactions, PFOA removal experiments evaluated the two base resin beads used for the production of A500P and A-860. The beads were donated by the manufacturer. It was assumed that since the beads were uncharged, any removal of PFOA by the resin beads should result from the hydrophobic interaction between PFOA molecules and the resin beads. This would then indicate the contribution of hydrophobic/hydrophilic interaction toward the overall uptake of PFOA. Figure 5 demonstrates that the uncharged styrenic and acrylic beads (base materials) of the two tested resins were unable to remove PFOA. Indeed, the very low BET surface area (<10 m^2^/g) and pore volume (<0.044 cm^3^/g) of the uncharged resin beads (Table 1) also support the observation of the negligible adsorption potential of PFCAs via hydrophobic interactions. Hence, it can be inferred that the dominant removal mechanism involves charge interactions between the negatively charged PFCA and the positively charged anion-exchange functional group.

Yu et al.^23^ observed a higher adsorption of PFOA onto the anion-exchange resins at pH 3 compared to pH 7. Thus, they indicated that the increased adsorption at the lower pH may have been due to the hydrophobic interaction between the uncharged species of PFOA and the resin. However, the p*K*_a_ of PFOA has been reported^25^ to be <1 and thus, PFOA is expected to be in its anionic form at pH 3. Hence, the increased adsorption of PFOA at pH 3 observed by Yu et al.^23^ may not necessarily have been due to the hydrophobic interaction between the neutral species of PFOA and the anion-exchange resin surface. Other previous studies have also suggested a minor role for hydrophobic adsorption during the removal of PFASs via IX processes.^20,41^

As discussed in Section 3.1, the current study along with several previous studies observed better performance of the polystyrenic resin structure as opposed to the polyacrylic resin structure during PFCA removal, which suggests that the hydrophobic (polystyrenic) composition of resins is more effective for PFCA removal. However, experiments with un-functionalized beads reveal negligible removal of PFCAs through hydrophobic interaction. This apparent contradiction could not be clarified as details of the functional groups on the IX resins are proprietary. Furthermore, it is unclear if the resin surface is altered once functional groups are attached to the resin base materials when manufacturing the IX resins.

## 4.0 Conclusions

The current study assessed the removal potentials of three selected PFCAs in UPW and GRW by ion exchange. In addition, it investigated the impact of the ion-exchange resin matrix (polyacrylic vs polystyrenic) on PFCA removal. To do so, the investigation was designed using two organic scavenger strong-base anionic resins, namely macroporous polystyrenic A-500P and microporous polyacrylic A-860. Both resins, with the exception of their base structure/matrix, had very similar properties, which enabled direct comparison between the removals achieved by the two resins. Two specific novel aspects of the current study are: (i) PFCA removal performance of the previously unreported A500P resin and its comparison with the well-studied A860; and (ii) the new GC–MS method that was developed for PFCA analysis in water. Under the conditions tested, the following conclusions can be drawn from the study:

- Depending on the resin dose, resin property, and natural water matrix, IX can be used to achieve simultaneous removal of NOM, inorganic anions, and PFCAs.
- NOM and inorganic anions substantially impacted PFCA removal in GRW, particularly in the case of the A-860 resin, which failed to achieve the removal of PFCAs despite having a higher resin dose compared to UPW experiments.
- Similar removal of DOC achieved by the two resins in GRW suggested that inorganic anions (sulfate in particular) were the dominant competitors for the anion-exchange resin A-860. Experiments in UPW confirmed that the presence of sulfate more severely affected PFCA removal for A-860 than for A-500P. Thus, if sulfate is present, particularly at elevated concentrations, utilities assessing treatment of perfluorinated compounds should consider this competition carefully when considering A-860-type ion-exchange resins for PFCA removal.
- The uncharged styrenic and acrylic beads (base materials) of the two tested resins were unable to remove PFOA, implying that the dominant removal mechanism involves charge interactions between the negatively charged PFCAs and the positively charged anion-exchange functional groups.
- Polystyrenic A500-P anion-exchange resin compared to polyacrylic A-860 exhibited higher adsorptive capacity and faster overall kinetics for the target PFCAs in UPW.

Future studies could explore whether combining the tested anion-exchange resins in series with granular-activated carbon treatment or even combining the two types of resins could enhance overall PFCA removal in natural water.

## Supporting Information Available

Properties of the target perfluoroalkyl carboxylic acids (PFCAs); properties of Grand River water; GC/MS analysis and method performance parameters; Freundlich isotherm parameters for selected adsorbents in ultrapure water; removal of target PFASs from Grand River water as a function of time illustrating reproducibility of removal trends; removal of selected inorganic anions from Grand River water as a function of time; and description of the GC/MS analytical method (PDF)
