# Supplementary material for: Ion-Exchange Treatment of Perfluorinated Carboxylic Acids in Water: Comparison of Polystyrenic and Polyacrylic Resin Structures and Impact of Sulfate on Their Performance
Source: ACS ES T Water. 2022 Jun 22;2(7):1195–205. doi: 10.1021/acsestwater.1c00501 (PMC9274775; doi:10.1021/acsestwater.1c00501)
Supplement: Supplementary file 1 — ew1c00501_si_001.pdf [file ew1c00501_si_001.pdf]

Supporting Information For:

Ion Exchange Treatment of Perfluorinated Carboxylic Acids in Water: Comparison of Polystyrenic and Polyacrylic Resin Structure and Impact of Sulfate on their Performance

M. Feisal Rahman[†§*], William B. Anderson[†], Sigrid Peldszus[†], and Peter M. Huck[†]

[†] Department of Civil and Environmental Engineering, University of Waterloo, 200 University Avenue West, Waterloo, ON, N2L 3G1, Canada.

[§] Living Deltas Hub, Dept. of Geography and Environmental Sciences, Northumbria University, Newcastle-upon-Tyne, NE1 8ST, United Kingdom. Email: mohammad.f.rahman@northumbria.ac.uk

*Corresponding Author

Table of Contents

No.	Title	Page#
**SI-A. Tables**		
Table SI-1	Properties of the target perfluoroalkyl carboxylic acids (PFCA)	3
Table SI-2	Properties of Grand River water	4
Table SI-3	Comparison of experimental conditions used in the current study with other published studies	5
Table SI-4	GC/MS analysis and method performance parameters	7
Table SI-5	Pseudo-second order kinetics model parameters in ultrapure water and Grand River water for the target PFCA for mixed-solute experiments (molar basis)	8
Table SI-6	Confidence intervals for pseudo-second-order kinetics model linear fitting parameters for ultrapure water and Grand River water kinetics experimental data	9
Table SI-7	Freundlich isotherm parameters for selected adsorbents in ultrapure water	10
Table SI-8	Comparison of DOC and inorganic anions removal observed during the current study with other published studies	11
**SI-B. Figures**		
Figure SI-1	Removal of DOC and various DOC fractions by anion exchange resins in Grand River water illustrating the reproducibility of NOM removal trends	12
Figure SI-2	LC-OCD Chromatograms of untreated Grand River water	13
Figure SI-3	LC-OCD Chromatograms of A-860 treated Grand River water	14
Figure SI-4	LC-OCD Chromatograms of A-500P treated Grand River water	15
Figure SI-5	Removal/exchange of selected inorganic anions from Grand River water as a function of time	16
**SI-C. Details of the GC/MS method developed for the current study**		
Figure SI-6	Optimized extraction protocol for PFCA analysis by GC/MS method developed for the current study	19
Figure SI-7	Summary of the derivatization process used for the developed GC/MS method	20
Figure SI-8	Characteristic chromatograms using µSIS (m/z: 131) of extracted and derivatized ultrapure water samples: A) blank; B) 0.05 µg/L PFCAs; C) 3.0 µg/L PFCAs, D) full scan mass spectra of PFNA	22
Table SI-9	Analysis and method performance parameters	23
**SI-D. References**		24

SI-A. Tables

Table SI-1. Properties of the target perfluoroalkyl carboxylic acids (PFCA)

Compound Name & CAS Registry #	Structure	[a]MW	log K_{OC} (L/kg)	Vapor Pressure (Pa)	Solubility (mg/L)
Perfluoroheptanoic acid (PFHpA) [375-85-9]	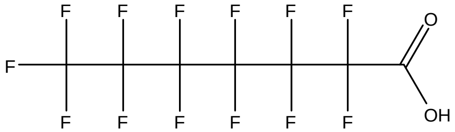	364.1		20.89[b] (25°C)	118,000[c] (21.6°C)
Perfluorooctanoic acid (PFOA) [335-67-1]	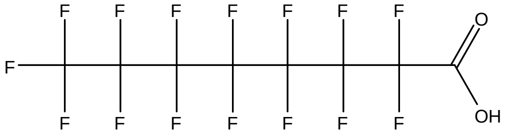	414.1	1.47[d]	4.17[b] (25°C)	4340[c] (24.1°C)
Perfluorononanoic acid (PFNA) [375-95-1]	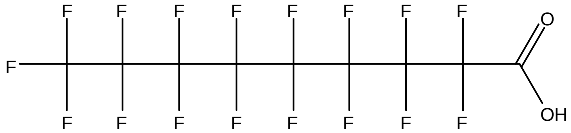	464.1	2.06 [d]	1.29[b] (25°C)	

a. United States National Library of Medicine 2011. ChemID plus database. Accessed 09/27 2013. http://chem.sis.nlm.nih.gov/chemidplus/.
b. Bhhatarai, B. and Gramatica, P. 2011. Prediction of aqueous solubility, vapor pressure and critical micelle concentration for aquatic partitioning of perfluorinated chemicals. Environ. Sci. Technol. 45(19), 8120-8128.
c. Kaiser, M. A., Barton, C. A., Botelho, M., Buck, R. C., Buxton, L. W., Gannon, J., Kao, C. C., Larsen, B. S., Russel, M. H., Wang, N., Waterland, R. L. 2006. Understanding the transport of anthropogenic fluorinated compounds in the environment. Organohalogen Compd. 68, 675-678.
d. Awad, E., Zhang, X., Bhavsar, S.P., Petro, S., Crozier, P.W., Reiner, E.J., Fletcher, R., Tittlemier, S.A. and Braekevelt, E., 2011. Long-term environmental fate of perfluorinated compounds after accidental release at Toronto airport. Environ. Sci. & Technol., 45(19), pp.8081-8089.

Table SI-2. Properties of Grand River water

Parameter	Collection Date	
	**03 February, 2014 (Set 1)**	**09 May, 2014 (Set 2)**
DOC (mg C/L)	5.0	4.7
Humic substances (mg C/L)	3.63	3.20
Biopolymers (mg C/L)	0.25	0.32
Building blocks (mg C/L)	0.63	0.58
UV_{254} (1/cm)	0.146	0.146
SUVA (L/mg C-m)	2.9	3.1
pH	8.20	8.50
Conductivity (µS/cm)	762	549
Alkalinity (mg/L as $CaCO_3$)	236	182
Turbidity (NTU)	1.3	5.4
Sulfate (mg/L)	29.3	16.6
Nitrate as nitrogen (mg/L)	3.7	3.0
Chloride (mg/L)	67.4	47.2

Table SI-3. Comparison of experimental conditions used in the current study with other published studies

Study	Kinetics Experiments	Isotherm Experiments
Rahman et al. 2022 (Current study)	○ Resin dose: in UPW 10 mg/L in UPW (0.045 mL/L) in Grand River water (GRW) 100 mg/L (0.45 mL/L)<br>○ Initial individual PFAS concentration: 0.003 mg/L<br>○ pH: 5.4–5.9 (UPW)/ 8.2–8.5 (GRW)<br>○ Time: up to 528 h	○ Resin dose: in ultrapure water (UPW) 0.5–12 mg/L (0.002–0.542 mL/L)<br>○ Initial individual PFAS concentration: 0.003 mg/L<br>○ pH: 5.4–5.9 (UPW)<br>○ Time: 252 h
Yu et al., 2009	○ Resin dose: 50 mg/L<br>○ Initial individual PFAS concentration: 50 mg/L<br>○ Initial pH: 3 or 7<br>○ Time: 168 h	○ Resin dose: 100 mg/L<br>○ Initial individual PFAS concentration: 20–250 mg/L<br>○ Initial pH: 3 or 7<br>○ Time: 168 h
Zagga et al. 2016	○ Resin dose: 1000 mg/L<br>○ Initial individual PFAS concentration: 1000 mg/L<br>○ Initial pH: 7.5<br>○ Time: 18 h stirred + 6 h rest + 120 h @ 20°C	
Kothawala et a. 2017	○ Resin dose: 10,000 mg/L<br>○ Initial individual PFAS concentration: 0.0025 mg/L<br>○ Initial pH: 7.5<br>○ Time: 15 min	
Dixit et al. 2019	○ Resin dose: 500 mg/L<br>○ Initial PFOA concentration: 0.01 mg/L<br>○ Initial pH: 7<br>○ Time: 2 min–24 h	○ Resin dose: 10–1000 mg/L<br>○ Initial individual PFAS concentration: 0.0001-0.1 mg/L<br>○ Initial pH: 7<br>○ Time: 24 h
Dixit et al. 2021	○ Resin dose: 0.4 mL/L<br>○ Initial individual PFAS concentration: 0.0001-0.01 mg/L<br>○ Initial pH: 7<br>○ Time: 2 min–24 h	○ Resin dose: 0.04–2.0 mL/L<br>○ Initial individual PFAS concentration: 0.0001-0.01 mg/L<br>○ Initial pH: 7<br>○ Time: 24 h
Del Moral et al. 2020	○ Resin dose: 0.25– 4.0 mL/L	

Study	Kinetics Experiments	Isotherm Experiments
	○ Initial individual PFAS concentration: 0.17– 0.65 mg/L<br>○ Initial pH: 8.0–8.1<br>○ Time: 24 h	
Park et al. 2020	○ Resin dose: 2–15 mL/L<br>○ Initial individual PFAS concentration: 0.0003 mg/L<br>○ Initial pH: 7<br>○ Time: 5 min–2 h	
Fang et al. 2021	○ Resin dose: 50 mg/L<br>○ Initial Individual PFAS concentration:<br>○ 0.00074–0.098 mg/L<br>○ Initial pH: 8.3<br>○ Time: 240 h	

Table SI-4. GC/MS analysis and method performance parameters

Name	MW of butyl ester	Qualification and quantitation ion (m/z)	Ultrapure water				Grand River water			
			MDL (ng/L)	LOQ (ng/L)	IP	Recovery (± RSD) (%)	MDL (ng/L)	LOQ (ng/L)	IP	Recovery (± RSD) (%)
PFHpA	420	100, ***131***, 169	23	74	4.6%	92.7 (4.0)	16	52	3.6%	108.1 (2.3)
PFOA	470	100, ***131***, 169	11	35	2.2%	115.2 (1.5)	20	65	3.4%	106.8 (2.8)
PFNA	520	100, ***131***, 169	16	51	1.9%	104.7 (2.4)	49	157	3.6%	95.7 (7.4)

IP- Instrument precision; MW-molecular weight; MDL- method detection level; LOQ- level of quantification; RSD - relative standard deviation: m/z in bold and italic are quantitation ions; N/A- data not available; n= 7 for MDL and LOQ calculations; n =8 for IP calculations.

Table SI-5. Pseudo-second order kinetics model parameters in ultrapure water and Grand River water for the target PFCA for mixed-solute experiments (molar basis)

Resins	q_e (mmol/g)	Exp. q_e (mmol/g)	q_e (mmol/g)	Exp. q_e (mmol/g)	q_e (mmol/g)	Exp. q_e (mmol/g)	k_2 (g. mmol^{-1}.h^{-1})			ϑ (mmol. g^{-1}.h^{-1})			R^2		
	PFHpA		PFOA		PFNA		PFHpA	PFOA	PFNA	PFHpA	PFOA	PFNA	PFHpA	PFOA	PFNA
**Ultrapure water (resin dose: 10 mg/L; individual target PFCA concentration: 3000 ng/L)**															
A-500P	0.00115	0.00102	0.00096	0.00087	0.00087	0.00080	30	48	60	4.1E-05	4.4E-05	4.5E-05	0.99	0.99	0.99
A-860	0.00079	0.00076	0.00073	0.00070	0.00077	0.00075	56	81	89	3.5E-05	4.4E-05	5.3E-05	0.99	0.99	0.99
**Grand River water (resin dose: 100 mg/L; individual target PFCA concentration: 3000 ng/L)**															
A-500P	0.00011	0.00010	0.00008	0.00008	0.00008	0.00008	1456	1604	1837	1.6E-05	1.2E-05	1.1E-05	0.99	1.00	1.00
A-860	0.00001	0.00001	0.00001	0.00001	0.000004	0.00001	865	1466	522	1.4E-07	1.0E-07	9.0E-09	0.89	0.71	0.02

Exp. q_e : experimental q_e

Table SI-6. Confidence intervals for pseudo-second-order kinetics model linear fitting parameters for ultrapure water and Grand River water kinetics experimental data

Adsorbent	$1/q_e$			$1/(k_2 q_e^2)$			R^2		
	**PFHpA**	**PFOA**	**PFNA**	**PFHpA**	**PFOA**	**PFNA**	**PFHpA**	**PFOA**	**PFNA**
**Ultrapure water (resin dose: ~10 mg/L; individual target PFCA concentration: ~3000 ng/L)**									
**A-500P**	0.0024 *(0.0020-0.0028)*	0.0025 *(0.0021-0.0029)*	0.0025 *(0.0022-0.0028)*	0.0028 *(0.0003-0.0053)*	0.0023 *(-4E-06-0.0045)*	0.002 *(0.0001-0.0039)*	0.99	0.99	0.99
**A-860**	0.0035 *(0.0034-0.0036)*	0.0033 *(0.0032-0.0034)*	0.0028 *(0.0027-0.0029)*	0.0033 *(0.0020-0.0046)*	0.0023 *(0.0008-0.0038)*	0.0017 *(0.0003-0.0030)*	0.99	0.99	0.99
**Grand River water (resin dose: ~100 mg/L; individual target PFCA concentration: ~3000 ng/L)**									
**A-500P**	0.0259 *(0.0267-0.0262)*	0.0282 *(0.028-0.0284)*	0.028 *(0.0278-0.0282)*	0.0071 *(0.0039-0.0102)*	0.0086 *(0.0062-0.0109)*	0.0082 *(0.0059-0.0106)*	0.99	1	1
**A-860**	0.2212 *(0.1126-0.3297)*	0.2988 *(0.0371-0.5604)*	0.5537 *(-4.723-5.831)*	0.8661 *(-0.4389-2.171)*	1.052 *(-2.094-4.198)*	11.18 *(-52.28-74.64)*	0.89	0.71	0.02

*Values in italic in parenthesis indicate 95% confidence intervals for the linear fitting parameters

**q_e is expressed in ng/mg and k_2 in mg/ng/d

Table SI-7. Freundlich isotherm parameters for selected adsorbents in ultrapure water

Compound	Freundlich intensity factor 1/n (dimensionless)		Freundlich capacity factor K_f $[(ng/mg)(L/ng)^{1/n}]$		R^2	
	A-860	A-500P	A-860	A-500P	A-860	A-500P
PFHpA	0.83 (0.57-1.10)	0.25 (0.07-0.44)	0.60 (-0.59-1.79)	229 (-50-507)	0.91	0.81
PFOA	1.96 (1.69-2.23)	0.33 (0.22-0.44)	<0.01 (-0.0002- <0.001)	108 (37-179)	0.99	0.92
PFNA	0.97 (0.35-1.6)	0.51 (0.35-0.68)	1 (-3-5)	41 (-5.3-88)	0.74	0.93

The 1/n and K_f values in parenthesis are 95% confidence intervals.

Table SI-8. Comparison of DOC and inorganic anions removal observed during the current study with other published studies

Study	*Water Characteristics*	*Resin dose*	*PFOA removal (after 24 h)*	*Removal of DOC (after 24 h)*	*Removal of inorganic anions (after 24 h)*
Dixit et al. 2020	o *SRNOM: 5 mg/L<br>o 30 mg/L of individual sulfate, nitrate, phosphate and bicarbonate ions<br>o pH: 7	o 10 mg/L of A-860 (0.05 mL/L)	Did not run PFOA experiments at this resin dose	~50%	o <10% removal of individual anions
		o 250 mg/L of A-860 (1.2 mL/L)	Did not run PFOA experiments at this resin dose	~80%	o Sulfate: 76%; Phosphate: 73%; Nitrate: 66%; Bicarbonate: 58%
		o 1000 mg/L of A-860 (4.5 mL/L)		~90%	o >99% removal of individual anions
Del Moral et al. 2020	o Ground water + SRNOM: 9.3 mg/L<br>o Chloride: 280 mg/L<br>o Sulfate: 94 mg/L<br>o Initial pH: 8.0–8.1<br>o Initial PFOA concentration: 0.3 mg/L	o 0.5 mL/L of A-860 (chloride form) (~100 mg/L**)	~40%	~64%	Did not report removal of anions
		o 0.5 mL/L of A-520 (chloride form)	~90%	~41%	
Rahman et al. 2022 (current study) (current study)	o DOC: 5 mg/L<br>o Sulfate: 29.3 mg/L<br>o Nitrate as nitrogen: 3.7 mg/L<br>o Chloride: 67.4 mg/L<br>o pH: 8.2<br>o Initial PFOA concentration: ~ 0.003 mg/L	o 100 mg/L of A-860 (0.45 mL/L**)	<10%	~65%	o Sulfate: 33%;<br>o Nitrate as nitrogen: 4%; Chloride: -19%
		o 100 mg/L of A-500P (0.45 mL/L***)	~78%	~35%	o Sulfate: 28%;<br>o Nitrate as nitrogen: 20%; Chloride: -16%

**SRNOM- Suwannee River natural organic matter; **mg/L to mL/L conversion based on Dixit et al. 2020*

SI-B. Figures

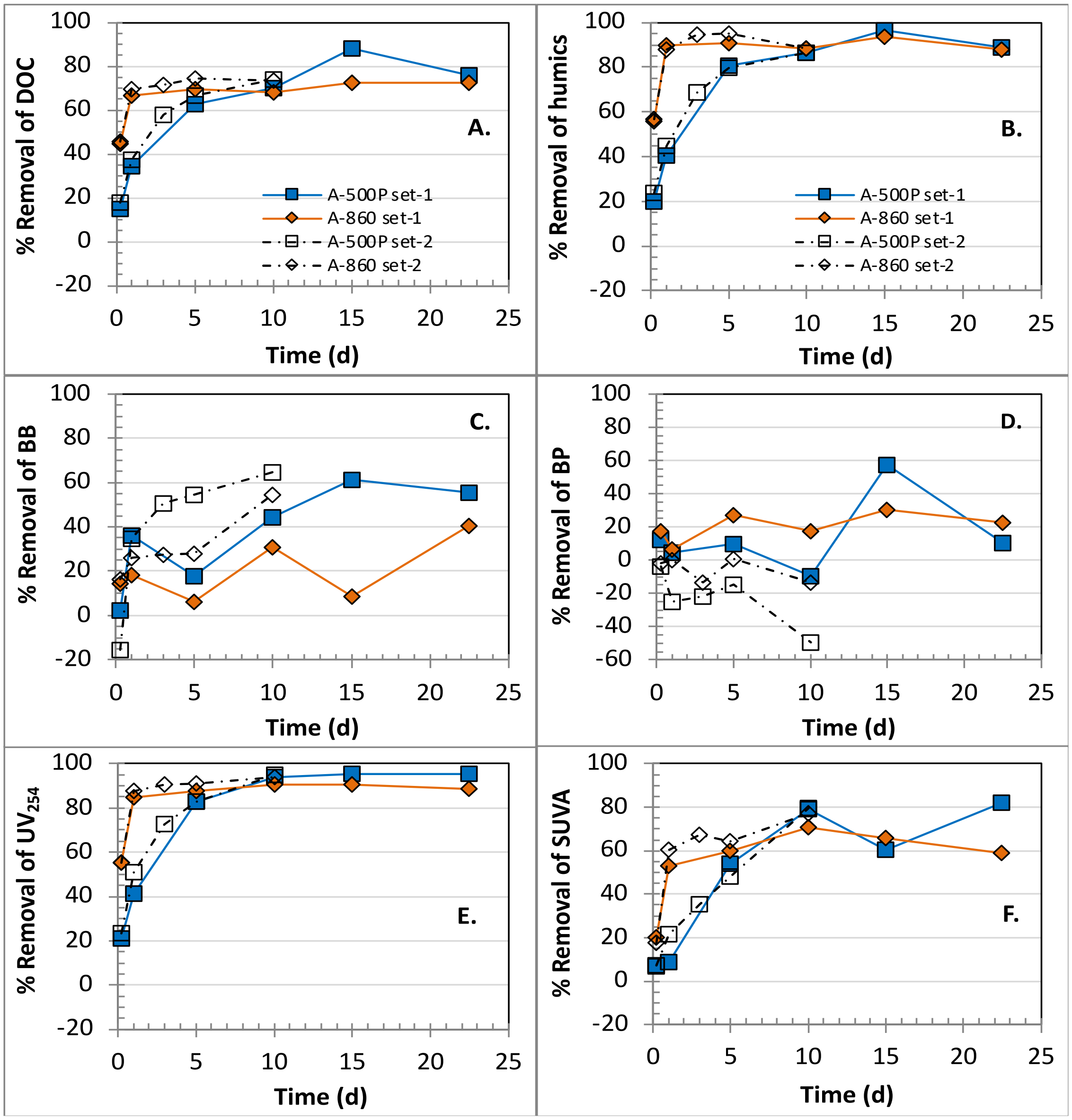

Figure SI-1. Removal of DOC and various DOC fractions by anion exchange resins in Grand River water (GRW) illustrating the reproducibility of NOM removal trends. Set 1 experiments were conducted on GRW collected in February 2012 and Set 2 experiments were conducted in GRW collected in May 2012. DOC: 5.0 mg/L for Set 1 and DOC: 4.7 mg/L for Set 2.

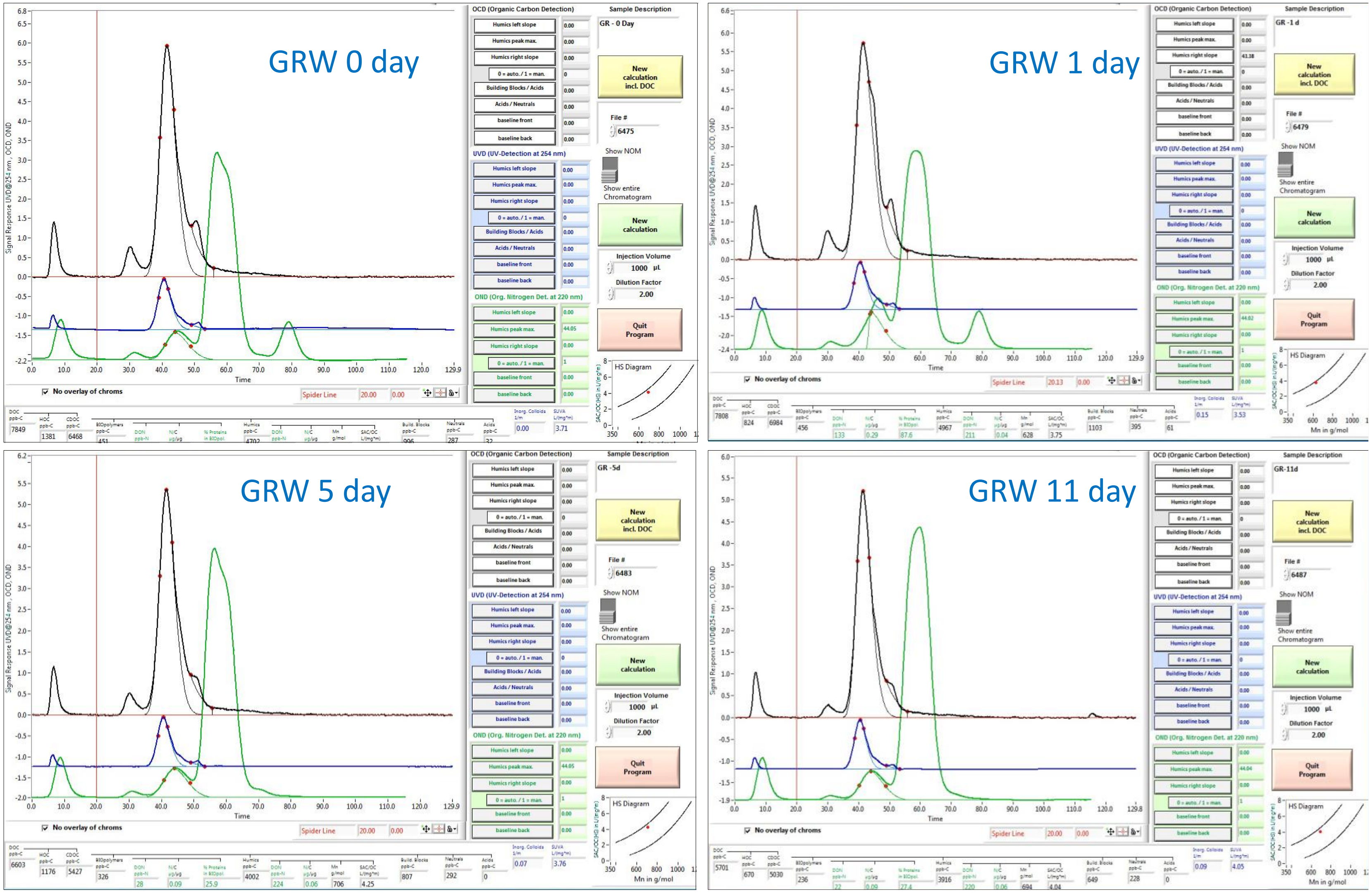

Figure SI-2. LC-OCD Chromatograms of untreated Grand River Water (GRW)

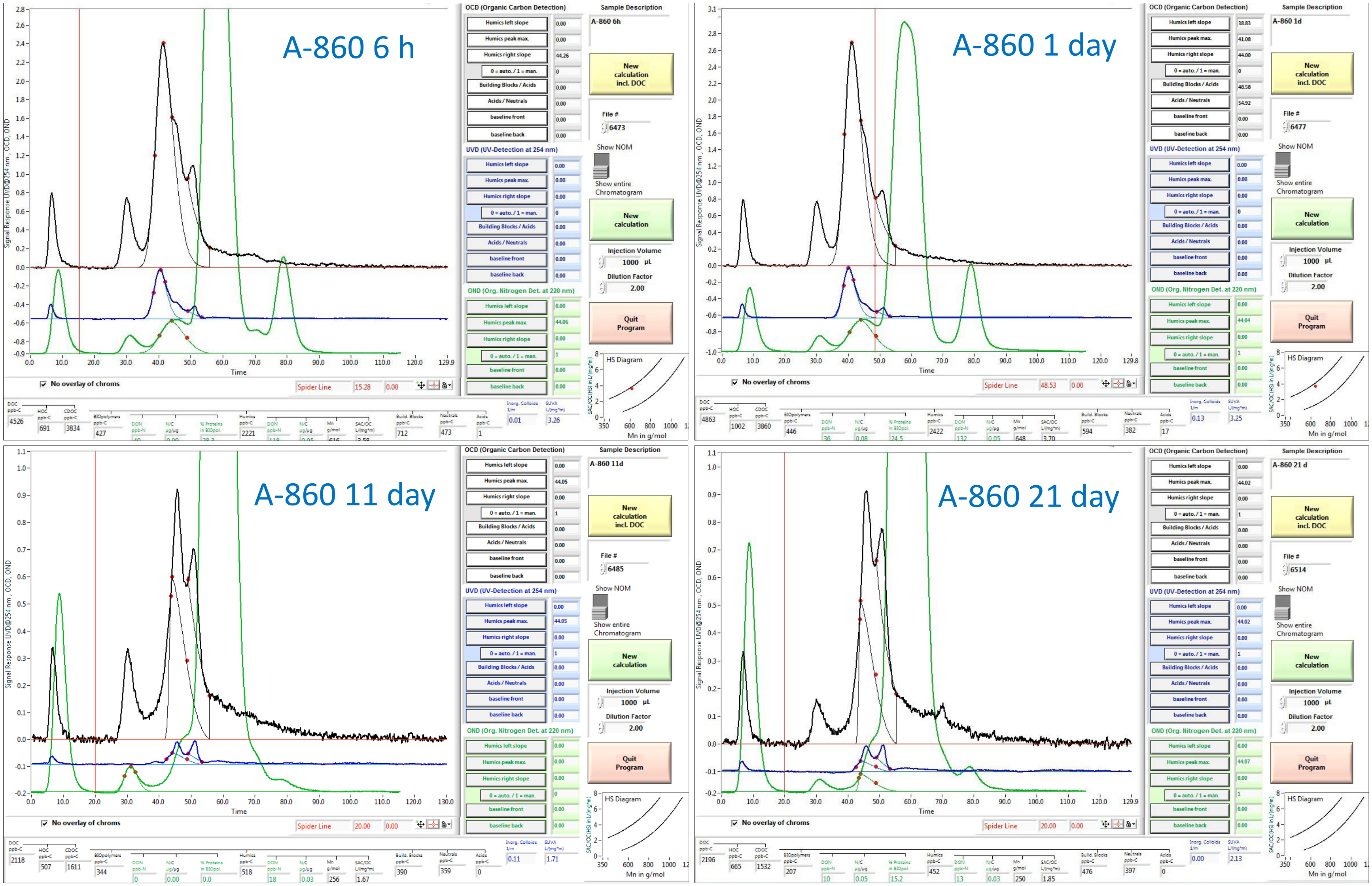

Figure SI-3. LC-OCD Chromatograms of Grand River water treated with A-860 resin

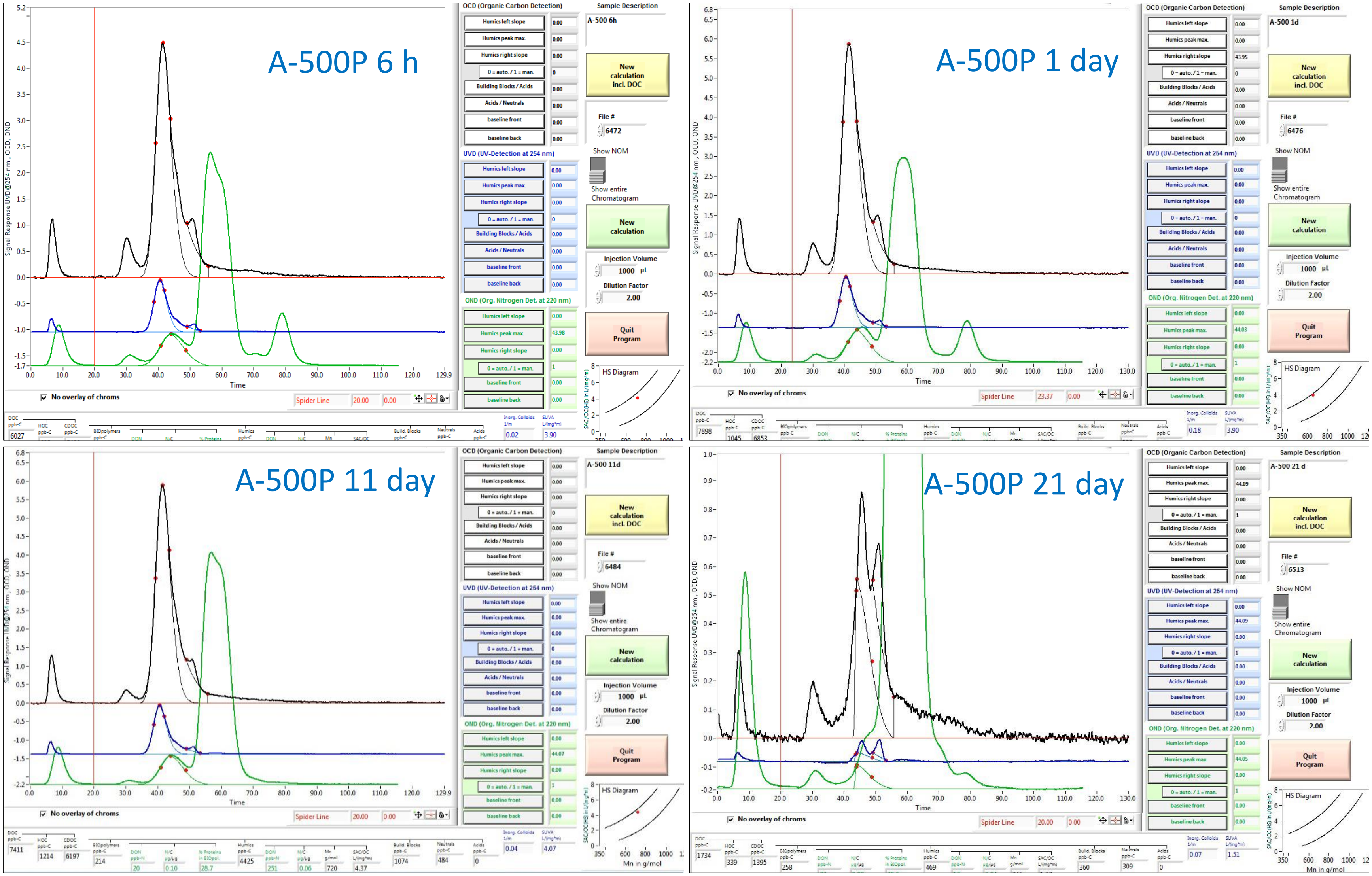

Figure SI-4. LC-OCD Chromatograms of Grand River water treated with A-500P resin

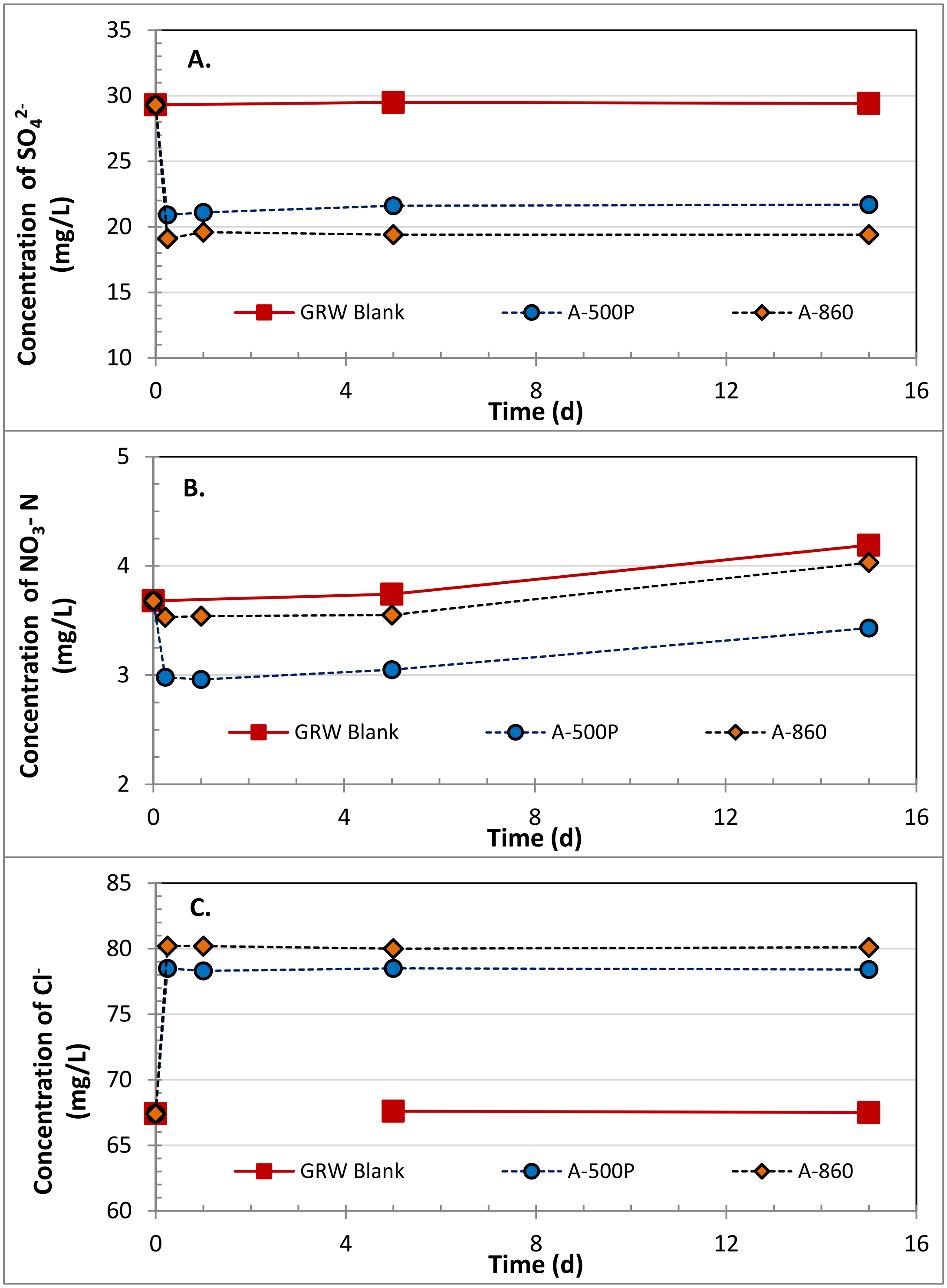

Figure SI-5: Removal/exchange of selected inorganic anions from Grand River water (GRW) as a function of time

Details of the GC/MS method developed for the current study

Overview

PFCA analysis is predominantly carried out using LC/MS. Only a limited number of GC/MS methods have been reported for analysis of select PFCs in aqueous and other matrices (Moody and Field 1999; Alzaga and Bayona 2004; Dufková et al. 2009, Dufková et al. 2012). However, many labs do not have access to LC/MS instrumentation and therefore, favor the more commonly available GC techniques. A gas chromatography-mass spectrometry (GC/MS) analytical method employing electron impact ionization was developed for the current study to simultaneously determine selected perfluorinated carboxylic acids (PFCAs) (C5-C9) concentrations in ultrapure and surface water samples. The target PFCAs were derivatized using butanol in the presence of sulfuric acid and heat. By employing central composite factorial design, the optimum derivatization reaction conditions were established. Details of the optimization can be found in Rahman (2015).

The method detection limits for PFCAs with six or more carbons were less than 31 ng/L in ultrapure water and less than 50 ng/L in surface water. Method recoveries for the target PFCAs were greater than 92% in both ultrapure water and surface water. Satisfactory levels (1.9%-5.1%) of instrument precisions (calculated by the relative standard deviation of eight injections of the same sample) were also achieved. The developed method was employed for analyzing selected target PFCAs for the subsequent bench-scale drinking water treatment study.

As is apparent from the description below, this GC/MS method is a lot more time consuming than LC-MS methods which are now routinely employed for PFAS quantification. LC-MS methods do not require a derivatization step, and they do not need an extraction step provided the MS has the necessary sensitivity.

Sample Preparation, Preservation and Background Contamination Prevention

Ultrapure water and surface water (collected from the Grand River, ON, Canada) were spiked with target PFCAs to establish calibration curves, method detection levels (MDLs), and levels of quantification (LOQ). Glass containers have been reported to irreversibly adsorb PFCAs (Martin et al. 2004) and hence, polypropylene (PP) containers and lab-ware were used whenever possible. Teflon®-based labware was also avoided to minimize potential background contamination.

Derivatization reactions were performed in 15 mL conical PP vials (VWR, West Chester, PA). All sample containers were washed thoroughly with ultrapure water, methanol, and ultrapure water three times each in sequence to avoid contamination. Sample containers were air dried prior to use. Water samples were passed through extraction cartridges in polypropylene transfer lines. Solvents and reagents were stored in vials covered by aluminum foil under their caps to minimize contamination from PTFE containing caps.

Solid Phase Extraction (Optimized Process)

Prior to derivatization, samples were concentrated using solid phase extraction pre-treatment. A previously published SPE method by Taniyasu et al. (2005) was adapted during the current study to accommodate GC/MS instrumentation. For Solid Phase Extraction (SPE) Oasis® HLB

(6 cc, 150 mg, 60 µm; hereafter referred to as HLB for hydrophilic–lipophilic balance) cartridges were purchased from Waters (Milford, MA).

Sample preconditioning, sample introduction, and elution steps were followed as described by Taniyasu et al. (2005). However, for the current GC/MS method 500 mL of sample instead of 100 mL was introduced to increase method sensitivity. In addition, eluted samples were blown down to dryness to facilitate the subsequent derivatization process by eliminating moisture and by swapping eluting solvent.

Briefly: prior to sample introduction HLB Cartridges were preconditioned using 5 mL methanol and then 5 mL of ultrapure water at about 2-3 drops/sec. Prior to extraction all samples were spiked with 150 µL of 1.96 mg/L internal standard solution ($^{13}C_8$-PFOA) prepared in methanol (corresponding to a final concentration of 0.588 µg/L in the sample). Spiked water samples (500 mL) were then passed through the conditioned cartridges at a rate of 1-2 drops/second. Effort was made to ensure that cartridges did not get dry at any time during preconditioning and sample introduction. Cartridges were then washed. For HLB cartridges, 5 mL of 5% methanol in ultrapure water was used as wash solution at 2-3 drops/sec. The cartridges were then dried thoroughly under vacuum to remove any excess water. The optimized extraction method is presented in Figure SI-6.

Once dried, HLB cartridges were eluted with 10 mL methanol. This was based on preliminary experiments conducted to optimize the eluent volume. The first 2 mL of eluent contained more than 65% of all the extracted PFCAs, and more than 99% were eluted within the first 6 mL. However, very small amounts of PFCAs could still be detected in the 6-8 mL and 8-10 mL eluates. Hence, for HLB cartridges a methanol volume of 10 mL was used to elute the extracted PFCAs.

The eluates were collected in 15 mL polypropylene vials and were then blown to dryness under a gentle stream of nitrogen at room temperature.

Preconditioning

- 5 mL of methanol at 2 drops/s
- 5 mL of milli-Q water at 2 drop/s

Sample introduction

- Water sample (500 mL) spiked with internal standard $^{13}C_8$-PFOA
    - For PFOA 150 µL of 1.96 mg/L standard
- Load the sample at 1 drop/s

Washing

- 5 mL of 5% methanol in milli-Q water
- Discard this fraction at 1 drop/sec

Elution, blow off and reconstitution

- Dry the cartridges to remove water
- Elute with 10 mL methanol
- Blow off the methanol extract to dryness with N_2 stream
- Reconstitute in 100 µL butanol

Derivatization

- Esterification of the perfluorinated acids; butanol in presence of H_2SO_4 to be used to form butyl esters of PFCAs
- Add Hexane as the primary solvent (BuOH: Hexane =1:4)

Figure SI-6. Optimized extraction protocol for PFCA analysis by GC/MS method developed for the current study

Derivatization (Optimized Procedure)

The residue resulting from the drying step was then reconstituted in 100 µL of anhydrous n-butanol which acted as the derivatizing reagent. The reconstituted extract solution was then stirred in a vortex mixer for 10-30 seconds. The derivatization reaction (Eq. S.1) took place under heat and acidic conditions. To provide acidic conditions, 10 µL of concentrated sulfuric acid (H_2SO_4) was added, stirred and capped. Vials were then heated at 50°C for three hours to form butyl esters of the PFCAs. The mixture was then allowed to cool down for approximately 20 min to room temperature and 100 µL of saturated Na_2CO_3 was added to neutralize the acid added previously. The mixture was then stirred in a vortex mixer for 10-30 seconds and allowed to settle for 3-5 minutes. Following the acid neutralization step, 400 µL n-hexane was added (final volume of the mixture 610 µL) to extract the PFCA butyl esters using liquid-liquid extraction. The mixture was stirred again in a vortex mixer for 10-30 seconds and was allowed to settle for 3-5 minutes. The upper hexane layer was collected for subsequent analysis by

GC/MS. The derivatized sample extract can be stored in refrigerator at 4°C up to 30 days. Summary of the derivatization process is presented in Figure SI-7.

$$RCOOH + C_4H_9OH \leftrightarrow RCOOC_4H_9 + H_2O \ldots\ldots (S.1)$$

R = $C_n F_{2n+1}$; Heat and H_2SO_4 were used as catalyst for the reaction

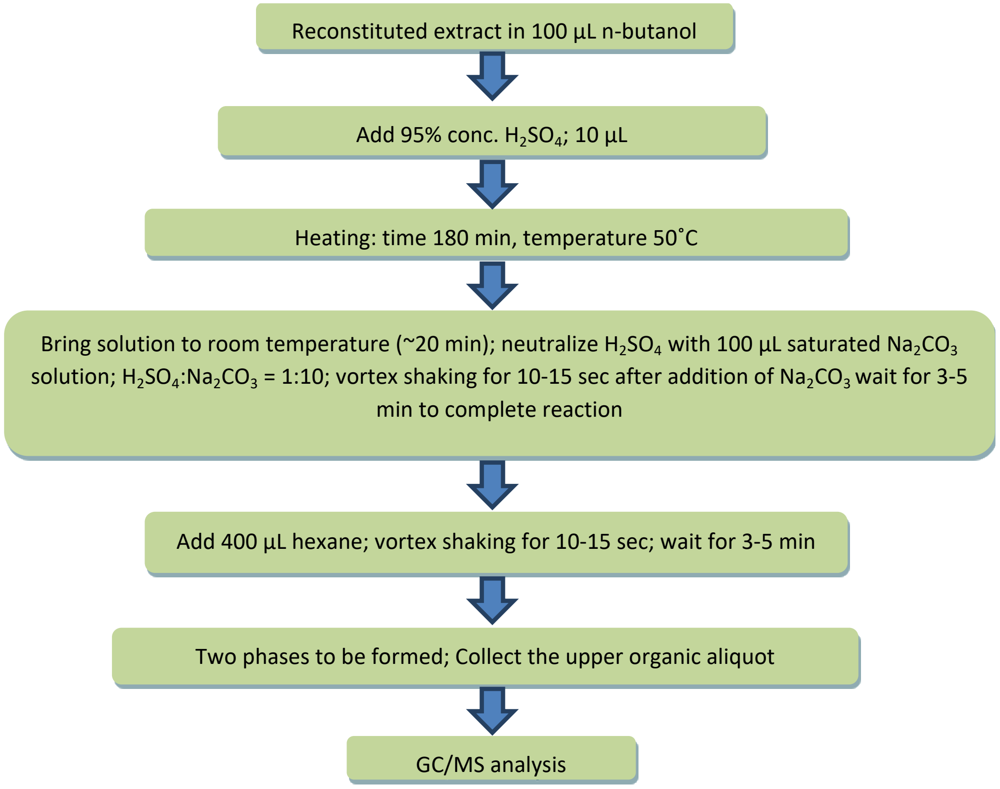

Figure SI-7. Summary of the derivatization process used for the developed GC/MS method

Instrumentation and Quantification

A Varian 3800® GC equipped with an 8210 Auto-sampler was used for all analyses. Helium was used as the carrier gas (constant flow at 1.0 mL/min). A DB-1701 fused silica column (30 m x 0.25 mm, 1.0 µm) coupled to a length of deactivated guard column was used for separation of the analytes. Injection of a 1 µL sample was performed with a split/splitless injector at a temperature of 250°C and held splitless for 1 min. Derivatized samples were kept at room temperature in the auto-sampler tray prior to analysis. The following temperature gradient program was used: 40°C for 5 min, 2°C /min to 50°C, 5°C/min to 120°C, 30°C/min to 240°C, keep for 5 min. Mass spectrometry was performed using a Varian 4000® MS in electron impact (EI) mode. Transfer line and ionization source temperatures were 250°C and 150°C, respectively. The solvent delay time was set to 12.5 min. The emission current was at 10 µamps. The MS was run in selected ion storage (µSIS) mode using the identification and quantification ions listed in Table SI-5. In preliminary experiments, mass spectra of the butylated derivatives were obtained in full scan mode, which were then used to determine the identification and quantification ions.

Identification and Quantification of PFCA with C≥ 5

Following derivatization with n-butanol and neutralization of the H_2SO_4, n-hexane was added to provide a non-polar phase and extract the formed PFCA butyl esters. n-hexane, having a lower boiling point (69°C) compared to n-butanol (117.4°C), eluted first in the chromatogram followed by an n-butanol peak. Consequently, earlier eluting butylesters of shorter chain PFCAs such as PFBA and PFPeA can overlapp with the n-butanol peak. It was observed that an n-hexane to butanol ratio of 4:1 could successfully be used to extract the derivatized butyl esters and separate PFCAs with five or more carbons from the butanol peak. An n-hexane to n-butanol ratio lower than that affected the separation of the shorter chain PFPeA and made the peak broader.

Example Chromatograms and Spectra, and QA/QC Results

Figure SI-8 presents GC/MS chromatograms for PFCAs with five or more carbons extracted with HLB cartridges at two different concentrations (0.5 and 3.0 µg/L) from ultrapure water samples and derivatized as described. Good response was achieved for all PFCA butylesters at 3 µg/L (Fig SI-8C) and even at 0.5 µg/L good response is evident for PFCA butylesters with carbon chain length of C6 and higher. The differences in retention time between each set of two consecutive PFCA butylester peaks are equidistant which is indicative of a homologous series.

When looking at mass spectra, the major EI fragmentation ions for PFCAs belong to two typical fragmentation series and differ by 50 amu corresponding to the mass of CF_2. One of the fragmentation series includes ions 69 $[CF_3]^+$, 119 $[C_2F_5]^+$, 169 $[C_3F_7]^+$, 219 $[C_4F_9]^+$ and the other series includes: 131 $[C_3F_5]^+$, 181 $[C_4F_7]^+$, 281 $[C_5F_9]^+$) (Alzaga and Bayona 2004; Moody and Field 1999; Dufková et al. 2009). In addition, other fragments (93$[C_3F_3]^+$ and 100 $[C_2F_4]^+$) have also been reported (Langlois et al. 2007). The full scan mass spectra of PFNA (Figure SI-5d) shows the presence of the characteristic fragmentation ions listed here. Three ions- m/z= 100, 131, and 169 were used as qualification ions, while m/z= 131 was used as the quantification ion for PFCAs with C ≥5. Previously Taniyasu et al. (2005) indicated that mass labeled 1,2 ^{13}C-PFOA can be used as a suitable internal standard for PFCAs with chain lengths between C6 and C10. Based on the conclusion drawn by Taniyasu et al. (2005) it was presumed that the recoveries of the mass labeled ^{13}C-PFOA (the internal standard for the current study) may also only be valid for chain length between C6 and C10 as the recoveries of the short chain PFCAs such as PFPeA and PFBA may differ from the longer chain PFCAs. Since PFPeA and PFBA were not used as target contaminants for the subsequent water treatment study, no quantitative work was performed on these PFCAs during this method development study.

In addition, PFBA could not be detected with the HLB cartridges using the current method due to the low extraction yield with the HLB cartridges as has been reported previously (Taniyasu et al. 2005).

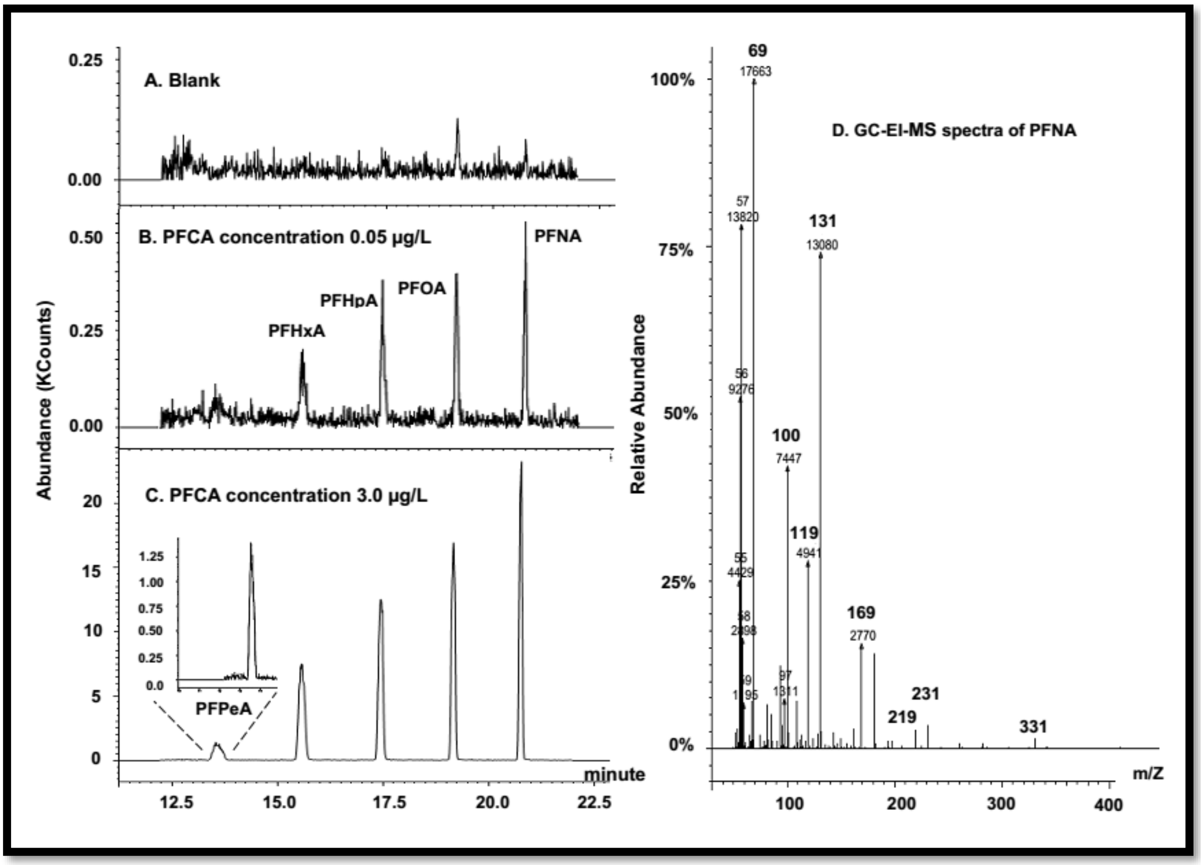

Figure SI-8: Characteristic chromatograms using µSIS (m/z: 131) of extracted and derivatized ultrapure water samples: A) blank; B) 0.05 µg/L PFCAs; C) 3.0 µg/L PFCAs, D) full scan mass spectra of PFNA.

The developed GC/MS method was successfully applied to analyze PFCAs spiked in ultrapure and surface water (Table SI-5). None of the target PFCAs were detected in unspiked ultrapure and surface water. Method recovery for the target PFCAs in ultrapure water ranged from 92% for PFHxA to 115% for PFOA and in surface water they ranged from 96% for PFNA to 108% for PFHpA. MDLs and LOQs were established in ultrapure and surface water for all target PFCAs except for PFBA and PFPeA. In ultrapure water the MDLs range from 11 ng/L to 30 ng/L and although similar, the MDLs of the PFCAs in surface water were somewhat higher. It was noted that MDLs and LOQs increased as the carbon chain length of PFCAs decreased which can be attributed to the decreased extraction yield of HLB cartridges as the carbon chain length decreased. Considering that this method applied EI ionization, the MDLs achieved using the method are satisfactory for conducting drinking water treatment studies for PFCA removal at trace concentrations. However, future studies can take advantage of negative chemical ionization to increase the sensitivity of the newly developed method.

The instrument precision limits were also determined for PFHxA, PFHpA, PFOA and PFNA (Table SI-5). The determined instrument precision limits for the GC/MS in both ultrapure water (1.9%-5.1%) and surface water (2.3%-3.6%) samples are very satisfactory.

Table SI-9. Analysis and method performance parameters

<table>
<tr><th rowspan="2">Name</th><th rowspan="2">MW of butyl ester</th><th rowspan="2">Qualification and quantification ion
(m/z)</th><th colspan="4">Ultrapure water</th><th colspan="4">Grand River water</th></tr>
<tr><th>MDL (ng/L)</th><th>LOQ
(ng/L)</th><th>IP</th><th>Recovery
(± RSD)
(%)</th><th>MDL
(ng/L)</th><th>LOQ
(ng/L)</th><th>IP</th><th>Recovery
(± RSD)
(%)</th></tr>
<tr><td>PFBA</td><td>270</td><td>100, 119, 169</td><td colspan="8" rowspan="2">N/A</td></tr>
<tr><td>PFPeA</td><td>320</td><td rowspan="5">100, 131, 169</td></tr>
<tr><td>PFHxA</td><td>370</td><td>30</td><td>95</td><td>5.1%</td><td>92.4 (5.2)</td><td>35</td><td>113</td><td>2.3%</td><td>107 (5.0)</td></tr>
<tr><td>PFHpA</td><td>420</td><td>23</td><td>74</td><td>4.6%</td><td>92.7 (4.0)</td><td>16</td><td>52</td><td>3.6%</td><td>108.1 (2.3)</td></tr>
<tr><td>PFOA</td><td>470</td><td>11</td><td>35</td><td>2.2%</td><td>115.2 (1.5)</td><td>20</td><td>65</td><td>3.4%</td><td>106.8 (2.8)</td></tr>
<tr><td>PFNA</td><td>520</td><td>16</td><td>51</td><td>1.9%</td><td>104.7 (2.4)</td><td>49</td><td>157</td><td>3.6%</td><td>95.7 (7.4)</td></tr>
</table>

IP- Instrument precision; MW-molecular weight; MDL- method detection limit; LOQ- limit of quantification; RSD - relative standard deviation: m/z in bold and italic are quantification ions; N/A- data not available; n= 7 for MDL and LOQ determinations; n =8 for IP determinations

SI-D REFERENCES

Alzaga, R. and Bayona, J. M. 2004. Determination of perfluorocarboxylic acids in aqueous matrices by ion-pair solid-phase microextraction-in-port derivatization-gas chromatography-negative ion chemical ionization mass spectrometry. J. Chromatography A 1042(1-2), 155-162.

Dixit, F., Barbeau, B. and Mohseni, M., 2020. Impact of natural organic matter characteristics and inorganic anions on the performance of ion exchange resins in natural waters. Water Supply, 20(8), pp.3107-3119.

Dufková, V., Cabala, R., Ševcík, V. 2012. Determination of C 5-C 12 perfluoroalkyl carboxylic acids in river water samples in the Czech Republic by GC-MS after SPE preconcentration. Chemosphere 87(5), 463-469.

Dufková, V., Cabala, R., Maradová, D., Štícha, M. 2009. A fast derivatization procedure for gas chromatographic analysis of perfluorinated organic acids. J. Chromatography A 1216(49), 8659-8664.

Langlois, I., Berger, U., Zencak, Z., Oehme, M. 2007. Mass spectral studies of perfluorooctane sulfonate derivatives separated by high-resolution gas chromatography. Rapid Commun. Mass Spectrom. 21(22), 3547-3553.

Moody, C. A. and Field, J. A. 1999. Determination of perfluorocarboxylates in groundwater impacted by fire-fighting activity. Environ. Sci. Technol. 33(16), 2800-2806.

Taniyasu, S., Kannan, K., Man, K. S., Gulkowska, A., Sinclair, E., Okazawa, T., Yamashita, N. 2005. Analysis of fluorotelomer alcohols, fluorotelomer acids, and short- and long-chain perfluorinated acids in water and biota. J. Chromatography A 1093(1-2), 89-97.

Yu, Q., Zhang, R., Deng, S., Huang, J. and Yu, G., 2009. Sorption of perfluorooctane sulfonate and perfluorooctanoate on activated carbons and resin: kinetic and isotherm study. Water Research, 43(4), pp.1150-1158.
